# Tissue-specific antitumor NK cell subsets identified in colorectal cancer liver metastases express candidate therapeutic targets

**DOI:** 10.1172/JCI190778

**Published:** 2025-10-28

**Authors:** Joanna Mikulak, Domenico Supino, Paolo Marzano, Sara Terzoli, Roberta Carriero, Valentina Cazzetta, Rocco Piazza, Elena Bruni, Paolo Kunderfranco, Alessia Donato, Sarah Natalia Mapelli, Roberto Garuti, Silvia Carnevale, Francesco Scavello, Elena Magrini, Jelena Zeleznjak, Clelia Peano, Matteo Donadon, Guido Costa, Guido Torzilli, Alberto Mantovani, Cecilia Garlanda, Domenico Mavilio

**Affiliations:** 1 Unit of Clinical and Experimental Immunology, IRCCS Humanitas Research Hospital, Rozzano, Milan, Italy.; 2 Unit of Experimental Immunopathology, IRCCS Humanitas Research Hospital, Rozzano, Milan, Italy.; 3 Department of Medical Biotechnology and Translational Medicine, University of Milan, Milan, Italy.; 4 Department of Biomedical Sciences, Humanitas University, Pieve Emanuele, Milan, Italy.; 5 Bioinformatics Unit, IRCCS Humanitas Research Hospital, Rozzano, Milan, Italy.; 6 Department of Medicine and Surgery, University of Milan-Bicocca, Monza, Italy.; 7 Unit of Innate Immunity in Inflammation and Cancer, IRCCS Humanitas Research Hospital, Rozzano, Milan, Italy.; 8 Center for Proteomics, Faculty of Medicine, University of Rijeka, Rijeka, Croatia.; 9 Institute of Genetics and Biomedical Research, National Research Council, Rozzano, Milan, Italy.; 10 Human Technopole, Milan, Italy.; 11 Department of Health Sciences, Università del Piemonte Orientale, Novara, Italy; Department of General Surgery, University Maggiore Hospital Della Carità, Novara, Italy.; 12 Unit of Hepatobiliary and General Surgery, IRCCS Humanitas Research Hospital, Rozzano, Milan, Italy.; 13 William Harvey Research Institute, Queen Mary University of London, London, United Kingdom.

**Keywords:** Immunology, Oncology, Immunotherapy, Liver cancer, NK cells

## Abstract

Liver metastases are relatively resistant to checkpoint blockade immunotherapy. The hepatic tissue has distinctive features including high numbers of NK cells. It was therefore important to conduct in-depth single-cell analysis of NK cells in colorectal cancer liver metastases (CRLMs) with an effort to dissect their diversity and to identify candidate therapeutic targets. By combining unbiased single-cell transcriptomic with multiparametric flow cytometry analysis, we identified an abundant family of intrahepatic CD56^bright^ NK cells in CRLMs endowed with antitumor functions resulting from specific transcriptional liver programs. Intrahepatic CD56^bright^ and CD56^dim^ NK lymphocytes expressed unique transcription factors (*IRF8*, *TOX2*), a high level of chemokines, and targetable immune checkpoints, including CXCR4 and the IL-1 receptor family member IL-1R8. CXCR4 pharmacological blocking and an anti–IL-1R8 mAb enhanced the effector function of CRLM NK cells. Targeting the diversity of liver NK cells and their distinct immune checkpoint repertoires is key to optimize the current immune therapy protocols in CRLM.

## Abstract 2

Disclosing the heterogenicity and theraputic targets of matched circulating and intrahepatic NK cells from patients undergoing surgical resection of liver metastatic lesions of colon cancer.

## Graphical abstract

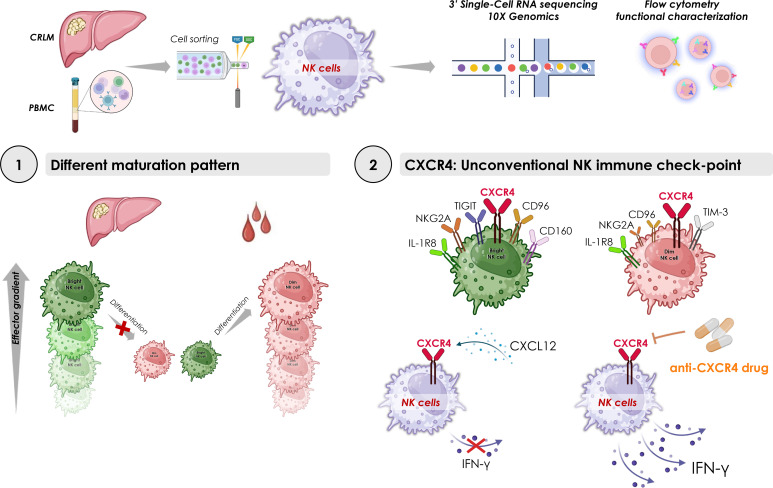

## Introduction

Colorectal cancer (CRC) is the third most frequent human malignancy and shows high rates of hepatic metastasis. Indeed, approximately 50% of CRC patients develop liver metastases (CRLMs) either as synchronous or metachronous disease (1, 2). Although the clinical outcome of CRLM remarkably improved over the past 20 years, we still lack a curative approach. The administration of neoadjuvant chemotherapies (na-CHTs) combined with biologic treatment followed by surgical resection represents the main available treatment, with a 5-year overall survival between 20% and 45% and a recurrence of 60% (3–5).

Several immune checkpoint (IC) inhibitors have been approved for clinical use and achieved encouraging results in a variety of solid tumors (6). However, CRLM patients showed poor clinical benefits from immunotherapies that included anti–PD-1/PD-L1 and/or anti-CTLA4 (5, 7–10), probably owing to the unique liver tumor microenvironment (11, 12). In addition, immunotherapeutic approaches are associated with favorable prognosis mainly in microsatellite instability–high/mismatch repair–deficient CRC tumors displaying high overall mutation burden, which represent about 3% of CRLMs (13–15). This is not the case for microsatellite-stable/mismatch repair–proficient CRC tumors (16–18). Therefore, exploring the heterogeneity of tumor-infiltrating immune cells and identifying cell type–specific ICs is key to identify the best responses to immunotherapies and to improve the clinical outcomes of CRLM patients.

The liver is considered a preferential tissue for natural killer (NK) cell residency. In humans, NK cells can reach up to 50% of intrahepatic lymphocytes and show a broad spectrum of functions by regulating the dynamic balance between immune tolerance and immune surveillance (19–22). Since their discovery in the mid-1970s, NK cells have been valued for rapid recognition and clearance of cancer cells in the absence of antigen specificity (23). Human NK cells are divided into 2 main subsets defined on the basis of their differential expression of CD56 and CD16, namely CD56^bright^CD16^–^ (CD56^bright^) and CD56^dim^CD16^+^ (CD56^dim^) NK cells (24, 25). Intrahepatic CD56^bright^ cells represent the major NK cell subset characterized by constitutive expression of the CXCR6 receptor and the tissue residency marker CD69 (26, 27). NK cell cytotoxicity and secretion of interferon-γ (IFN-γ) are controlled by a dynamic balance exerted by an array of inhibitory receptors (iNKRs) and activating receptors (aNKRs) differentially expressed on the cell surface of NK cells (28).

Several studies reported the presence at high frequencies of tumor-infiltrating NK cells within primary and metastatic liver malignancies, including hepatocellular carcinoma and CRLMs (22, 29–32). However, the relevance and clinical impact of tissue-resident NK cell immune surveillance against CRLMs are still largely unknown and being debated (21).

The present study had the aim of dissecting the functional heterogeneity of matched circulating and intrahepatic NK cells isolated from CRLM patients undergoing surgical resection of metastatic lesions, as well as investigating the expression of ICs and other potential targets of immunotherapy. By combining single-cell transcriptional analysis of NK and CD45^+^ cells with multiparametric flow cytometry and functional assays, we show here that intrahepatic CD56^bright^ NK cells in CRLMs are mature and potent tissue-resident antitumor effectors expressing a unique transcriptional profile that makes them distinguishable from their circulating counterparts and from both peripheral and intrahepatic CD56^dim^ NK cells. Moreover, the liver metastatic niche is characterized by the expression of ICs on several NK cell subsets. Among these, we focused on the IL-1 receptor family member IL-1R8, which we previously identified as an IC that negatively regulates IL-18–dependent NK cell activation, involved in resistance to hepatic carcinogenesis and hematogenous CRLM (31). We found that IL-1R8 was ubiquitously expressed by intrahepatic NK cells, and its targeting with a blocking monoclonal antibody (mAb) improved NK cell antitumor effector functions. In addition, CXCR4 emerged as a potential therapeutic target, as suggested by the increased NK cell production of IFN-γ after its blocking with the antagonist plerixafor.

## Results

### Dissection of intrahepatic NK cell heterogeneity in CRLM by single-cell RNA sequencing.

**Figure 1:**
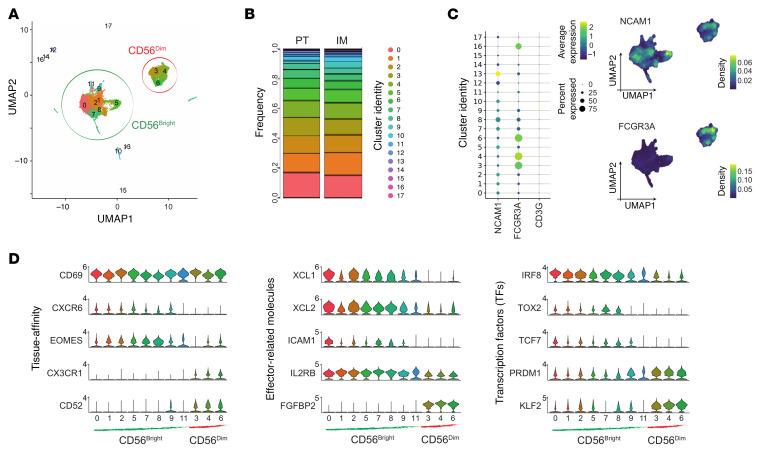
Single-cell RNA-Seq analysis of liver NK cells from invasive margin and peritumor CRLM tissue sites. (**A**) UMAP projection of liver NK (NK_LR_) cells from invasive margin (IM; *n* = 9,208) and peritumor (PT; *n* = 14,091) of 3 CRLM patients. CD56^bright^ clusters are circled in green, CD56^dim^ in red. (**B**) Bar plot showing relative frequency of clusters in IM and PT (normalized per tissue). (**C**) Left: Dot plot of *NCAM1*, *FCGR3A*, and *CD3G* expression. Right: Kernel density of *NCAM1* and *FCGR3A* on UMAP plot. (**D**) Violin plots of selected genes across NK_LR_ cell clusters, ordered according to CD56^bright^ or CD56^dim^.

To decipher the heterogeneity of intrahepatic NK cells, single-cell RNA sequencing (scRNA-Seq) was performed. FACS-sorted NK cells (Lin^–^CD56^+^, excluding CD3^+^, B, and myeloid cells) were isolated from CRLM and peripheral blood (PB) samples from 3 patients (2 male and 1 female) undergoing limited surgical liver resection for synchronous CRLM disease after na-CHT treatment (Supplemental Figure 1, A and B; supplemental material available online with this article; https://doi.org/10.1172/JCI190778DS1). Samples were collected from the peritumor (PT) and invasive margin (IM) areas of CRLM. The integrated transcriptomic profiles of 14,091 PT and 9,208 IM liver NK (NK_LR_) cells were interrogated by nonlinear dimension reduction with uniform manifold approximation and projection (UMAP) (Figure 1A and Supplemental Figure 1C). In total, 18 liver cell clusters (c_LR_) were obtained. PT and IM NK_LR_ cells were equally distributed among clusters with minor differences between individual patients (Figure 1B and Supplemental Figure 1D) and were thus merged for subsequent analyses. Contaminating clusters enriched in CD3^+^ lymphocytes and myeloid and B cells (c10_LR_, c12–17_LR_) represented less than 0.07% of total events and were excluded from further analysis (Figure 1C and Supplemental Figure 1E).

Unsupervised analysis subdivided NK_LR_ cells into two CD56^dim^ and CD56^bright^ macro-clusters that differed in the expression of CD16 (*FCGR3A*) and CD56 (*NCAM1*) (Figure 1C, Supplemental Figure 1F, and Supplemental Table 2) (33, 34). CD56^dim^ and CD56^bright^ NK_LR_ cells included 3 clusters (c3–4_LR_, c6_LR_) and 8 clusters (c0–2_LR_, c5_LR_, c7–9_LR_, c11_LR_), respectively. As expected, the majority of the NK_LR_ fraction (69%) was represented by CD56^bright^ cells (Figure 1, A and B) (26). Differentially expressed gene (DEG) analysis confirmed the liver-residency profile of CD56^bright^ NK_LR_ cells, characterized by coexpression of *CXCR6*, *EOMES*, and *CD69* and lack of *CD52* (Figure 1D and Supplemental Figure 2) (21). In addition, CD56^bright^ NK_LR_ cells shared high expression of the chemokines *XCL1* and *XCL2*, the cytokine receptor *IL2RB*, the costimulatory molecule *ICAM1*, and a peculiar coexpression of transcription factors (TFs) both inhibiting (*TCF7*) and promoting (*IRF8*, *TOX2*) NK cell maturation (35–39). On the other hand, CD56^dim^ NK_LR_ cells expressed *FGFBP2*, the memory NK cell marker *CD52*, and TFs such as *PRDM1*, *KLF2*, and the *CX3CR1* receptor, which is involved in NK cell migration from PB to peripheral tissues (40–42), thus suggesting they are not a resident population (Figure 1D). Although we cannot entirely exclude the presence of intravascular NK cells, given the highly perfused nature of the liver, the transcriptional profile of CD56^dim^ NK_LR_ cells strongly suggests peripheral recruitment or transient retention.

**Figure 2:**
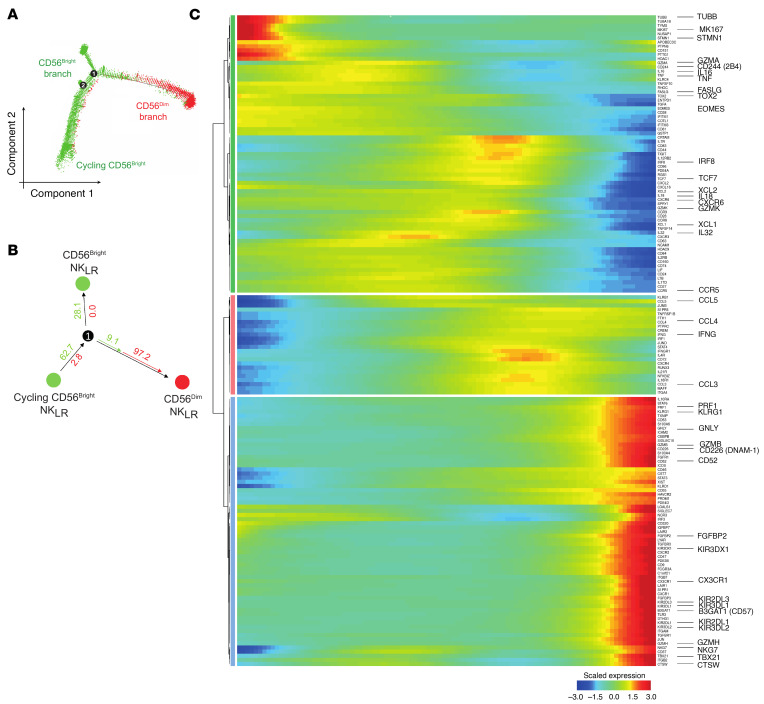
Pseudotime analysis of CD56^bright^ and CD56^dim^ NK_LR_ cells. (**A**) Monocle2 pseudotime trajectory of NK_LR_ cells. CD56^bright^ and CD56^dim^ cells are shown in green and red, respectively. (**B**) Relative frequency of CD56^bright^ (green) and CD56^dim^ (red) cells across branches, normalized within each branch. (**C**) Heatmap of selected driver genes along pseudotime. Cells are ordered left to right; colors indicate scaled (*z* score) expression.

Pseudotime analysis was applied to unravel the developmental trajectories between NK_LR_ cell clusters in CRLM. The pseudotime results by Monocle2 showed CD56^bright^ and CD56^dim^ cells along 3 separated branches (Figure 2, A and B, and Supplemental Figure 3A). 97.2% of CD56^dim^ NK cells were located on the right branch; in contrast, the majority of CD56^bright^ NK cells were distributed among the left (62.7%) and upper (28.1%) branches (Figure 2B). Importantly, we observed a minimal developmental relationship between CD56^bright^ and CD56^dim^ NK cells (9.1%) (Figure 2B), suggesting that a direct transition between the 2 subsets may be less evident in this setting. To validate this observation, pseudotime trajectories were also inferred using the Slingshot algorithm (Supplemental Figure 3B). This analysis confirmed the relative independence of intrahepatic CD56^bright^ and CD56^dim^ NK cells and highlighted the enrichment of CD56^bright^ clusters with distinct functional profiles within the CRLM immune environment.

Highly proliferative cells in CD56^bright^ c8_LR_ and c7_LR_ expressing hallmark genes of DNA replication (e.g., *TUBB*, *MIKI67*, *STMN1*) were located at the starting root node of the CD56^bright^ cell branch (Figure 2, A–C, and Supplemental Figure 3, A and C). These were associated with a liver tissue-residency profile, as suggested by the expression of *EOMES* and *CXCR6*, and terminal maturation markers such as *TOX2* and *CD244* (Figure 1D, Figure 2C, and Supplemental Figure 3C), indicating their high transcriptional dynamics. From these, we observed a progression toward a full effector stage following the CD56^bright^ cell fate of c0–2_LR_, as suggested by pseudotime-dependent DEGs including cytokines (e.g., *XCL1*, *IL16*, and *TNF*), effector function molecules (e.g., *GZMB*, *GZMK*, and *PRF1*), and TFs (e.g., *EOMES* and *TOX2*) (Figure 2C and Supplemental Figure 3C). Finally, we further identified CD56^bright^ c9_LR_ and c11_LR_ clusters, characterized by more advanced pseudotime positions, showing high expression of adaptive-like (*KLRC2*, *CD3E*) (43) and mitochondrial-active (e.g., *MTRNR2L1*/*8*/*12*/*18*) signature genes, respectively (Figure 1D, Figure 2C, Supplemental Figure 2, and Supplemental Figure 3, A and C).

On the other hand, CD56^dim^ NK cells showed lower transcriptional heterogeneity as highlighted by the expression of terminally mature NK cell markers (*KIR*s, *B3GAT1*, *CD52*, *TBX21*, *KLRG1*, *FGFBP2*, *NKG7*, *CD226*), cytotoxic mediators (*GZMB*, *GZMH*, *PRF1*, *GNLY*, *CTSW*), and the tissue-homing receptor *CX3CR1* (Figure 1D, Figure 2C, and Supplemental Figure 2).

Overall, pseudotime analysis revealed that intratumoral hepatic CD56^bright^ and CD56^dim^ NK cells followed largely independent trajectories with minimal overlap, with CD56^bright^ cells displaying multiple intermediate states, whereas CD56^dim^ cells predominantly occupied terminally differentiated states with limited heterogeneity.

### Functional status and immune checkpoint profile of liver NK cells in CRLM.

**Figure 3:**
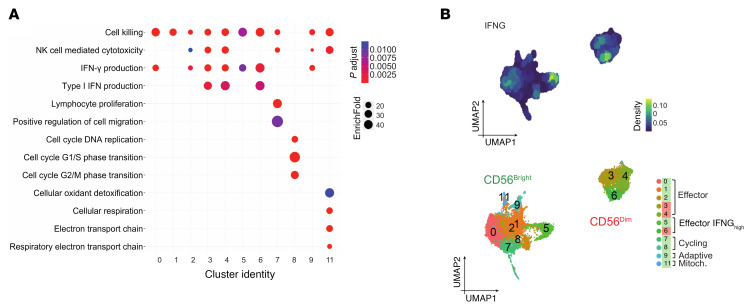
Functional enrichment and *IFNG* expression in NK_LR_ cells. (**A**) Dot plot of GO biological process terms (*q* ≤ 0.01) enriched in intrahepatic NK_LR_ cell clusters [DEGs: FDR ≤ 0.05, log(fold change) > 0.25]. Dots are colored by EnrichFold and sized by adjusted *P* value. (**B**) Kernel density of *IFNG* expression on the UMAP plot (top). The clustering of cells is shown at the bottom, with clusters grouped according to their transcriptional features.

**Figure 4:**
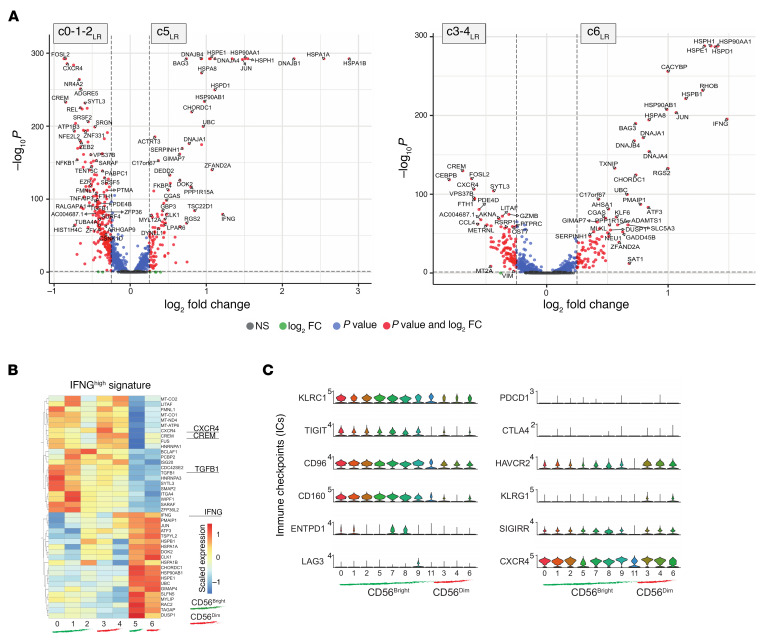
Comparison of liver CD56^bright^ and CD56^dim^ NK_LR_ cells. (**A**) Volcano plot of DEGs in CD56^bright^ c5_LR_ versus c0–1–2_LR_ (left) and CD56^dim^ c6_LR_ versus c3–4_LR_ (right). (**B**) Heatmap of *IFNG^high^* signature gene expression across NK_LR_ clusters. (**C**) Violin plots of selected immune checkpoint genes.

We then performed Gene Ontology (GO) enrichment analysis of DEGs for biological processes to elucidate pathways associated with intrahepatic NK cell clusters. GO enrichment analysis highlighted a strong association of NK_LR_ cells with “cell killing” (Figure 3A). “NK cell–mediated cytotoxicity” mainly correlated with the CD56^dim^ transcriptomic profile. Cell cycle and proliferation pathways were associated with c7_LR_ and c8_LR_ CD56^bright^ clusters, whereas “cellular oxidant detoxification” and electron transport chain pathways were enriched in c11_LR_, as marked by mitochondrial genes. “Type I IFN production” was exclusively observed in CD56^dim^ cells, whereas pathways of IFN-γ production were shared by CD56^bright^ and CD56^dim^ NK_LR_ cells. In particular, we identified 2 clusters, CD56^bright^ c5_LR_ and CD56^dim^ c6_LR_, associated with a distinctive *IFNG^high^* transcriptomic profile (Figure 3B), suggesting their effector activation. *IFNG^high^* cell subsets showed higher expression of heat shock proteins (HSPs) (e.g., *HSPA1A*, *HSPA1B*, *HSPB1*), chaperone and co-chaperone for HSP (*DNAJA1*, *CHORDC1*), and signaling molecules such as *JUN*, *GIMAP4*, and *DUSP1* (Figure 4, A and B). Similar results were observed both in PT and IM compartments (Supplemental Figure 4A).

To decipher the mechanisms that regulate these *IFNG-*expressing cells conserved across CD56^bright^ and CD56^dim^ NK_LR_, we investigated the expression of ICs. CD56^bright^ and CD56^dim^ cell subsets were distinguished for their IC pattern (Figure 4C). *KLRC1*, *TIGIT*, *CD96*, and *CD160* were mainly associated with the CD56^bright^ phenotype. In addition, *ENTDP1* (CD39) was expressed by cycling CD56^bright^ cells in c7–8_LR_. *LAG3* identified adaptive-like (c9_LR_) CD56^bright^ cells. In contrast, CD56^dim^ cells were marked by *HAVCR2* (TIM-3) and *KLRG1*, and higher expression of specific *KIR*s (Supplemental Figure 4B). We found low expression of *PDCD1* (PD-1) and *CTLA4* in all NK_LR_ cells. Notably, *SIGIRR* (IL-1R8), which we previously identified as an NK cell IC (31), was ubiquitously expressed by all NK_LR_ cell clusters. In addition, lower expression of *CXCR4*, an unconventional IC of liver NK cells (44), was observed in the *IFNG^high^* c5_LR_ and c6_LR_ (Figure 4C), as also shown in the heatmap (Figure 4B).

**Figure 5:**
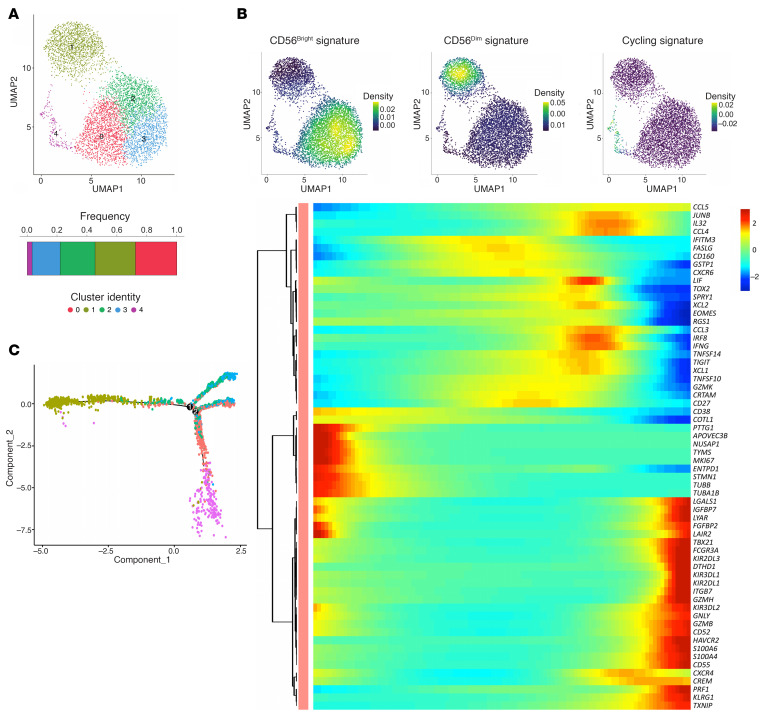
Healthy hepatic NK cells. (**A**) Top: UMAP projection of integrated NK cells (*n* = 5,760) from 5 healthy donors. Bottom: The bar plot shows the cluster frequencies normalized on total cells. (**B**) Kernel density of CD56^bright^, CD56^dim^, and cycling signature scores on UMAP. (**C**) Left: Pseudotime trajectory of hepatic NK cells (colored by cluster). Right: Heatmap of selected driver genes ordered by pseudotime, with scaled (*z* score) expression shown by color.

To precisely define the contribution of CRLM microenvironment to the orientation of intrahepatic NK cells, we harmonized scRNA-Seq data from healthy liver samples (45) (see Methods). In the healthy liver, we identified CD56^bright^, CD56^dim^, and cycling NK cell subsets, with a clear separation between the CD56^bright^ and CD56^dim^ populations (Figure 5, A and B, and Supplemental Table 2) (26, 33, 34). This distinction mirrors what we observed in CRLM samples, as illustrated by UMAP embedding annotated with phenotype-specific gene signature scores (Figure 5B).

As in the pathological condition, pseudotime analysis in healthy liver also revealed only a minimal developmental connection between CD56^bright^ and CD56^dim^ NK cells (Figure 5C), with cycling NK cells located at the starting root of the trajectory. However, when we projected the pathological pseudotime ordering genes (Figure 2C) onto the healthy dataset (Figure 5C), many of them were absent (e.g., *GZMA*, *TNF*, *CD244* [2B4], *IL16*, *PRF1*), indicating that the pathological milieu shapes the developmental trajectory of NK cells.

**Figure 6:**
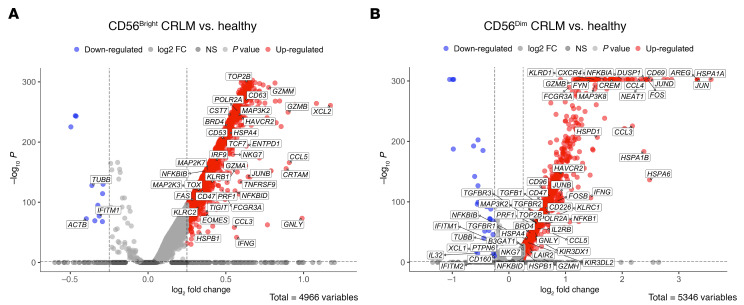
Comparison of CD56^bright^ and CD56^dim^ NK cells in CRLM versus healthy liver. Volcano plots of DEGs for CD56^bright^ clusters (**A**) and CD56^dim^ clusters (**B**) in CRLM versus healthy liver.

To investigate the NK cell subset–specific transcriptional changes induced by CRLM, we performed DEG analysis (Figure 6, A and B). Volcano plots highlighted that both CD56^bright^ and CD56^dim^ NK cells exhibited features of activation, including the upregulation of transcriptional programs mediated by AP-1 and NF-κB, as well as activation of MAPK signaling pathways. In both subsets, we observed increased expression of *IFNG*, cytotoxic effectors (*GZMB*, *GNLY*, *PRF1*), activating receptors (*FCGR3A*, *NKG7*), and transcriptional regulators such as *TOP2B*, *BRD4*, and *POLR2A*. Notably, CD56^bright^ NK cells showed elevated expression of effector-associated genes such as *EOMES*, *FAS*, *TOX*, *CRTAM*, and *TNFRSF9*. Similarly, CD56^dim^ NK cells upregulated the effector-associated gene *CD226* (DNAM-1) and differentiation markers such as *B3GAT1* (CD57) along with *KIR*s and *TGFB1*, *TGFBR2*, and *TGFBR3*, important regulators of NK cell activity (46). We also observed changes in the expression of immune checkpoint molecules: CD56^bright^ NK cells expressed higher levels of *TIGIT* and *ENTPD1*, while CD56^dim^ NK cells upregulated *KLRC1* and *LAIR2*, supporting our findings of subset-specific immune regulation (Figure 4C).

Together, these data demonstrate that CRLM profoundly reshapes the transcriptional landscape of both CD56^bright^ and CD56^dim^ NK cells, fostering an effector-like phenotype marked by a strong *IFNG* signature alongside finely tuned immune checkpoint modulation. While both subsets exhibit activation features, they preserve their distinct transcriptional and phenotypic identities, challenging the notion that effector functions in intrahepatic NK cells are confined to the CD56^dim^ population.

### Ligand-receptor interactions shaping NK_LR_ crosstalk in CRLM.

**Figure 7:**
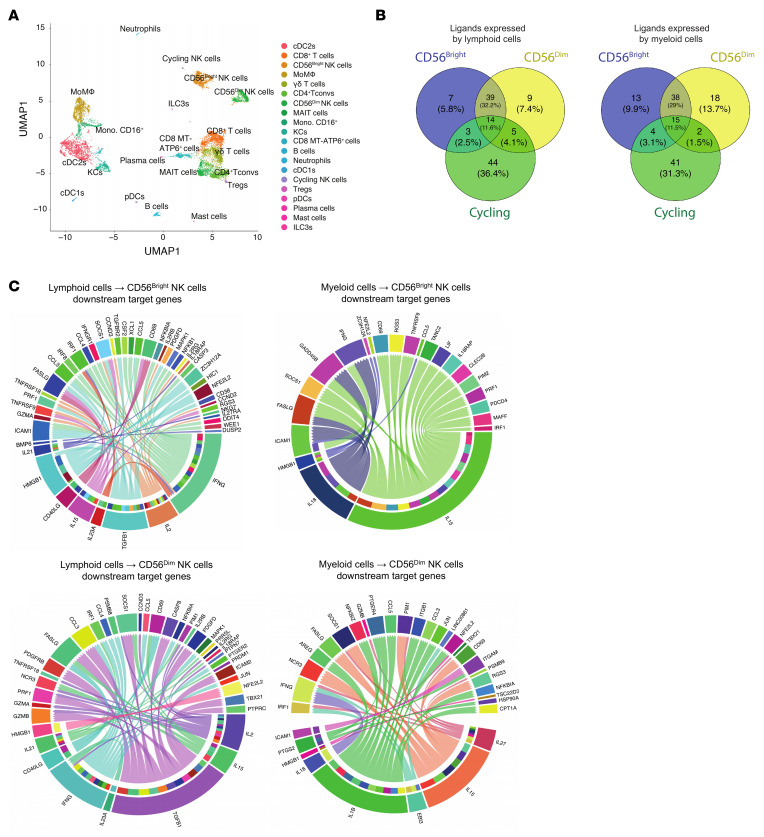
Intercellular communication in the immunological milieu of CRLM. (**A**) UMAP projection of intrahepatic CD45^+^ cells colored by cell family. (**B**) Venn diagrams of common and specific ligands from lymphoid (left) and myeloid (right) cells interacting with CD56^bright^, CD56^dim^, and cycling NK cells. (**C**) Chord plots of the top 100 predicted interactions (NicheNet) for CD56^bright^ (top) and CD56^dim^ (bottom) NK cells, ranked by regulatory potential. In each circular plot, one arc contains the ligands (derived from lymphoid [left] or myeloid [right] cells) and the opposite arc contains the downstream NK target genes. Directional chords connect each ligand to its predicted NK target genes, with the pointed end of each chord indicating the direction of regulation. Bars above the ligand arc represent the proportion of downstream target genes regulated by each ligand.

**Figure 8:**
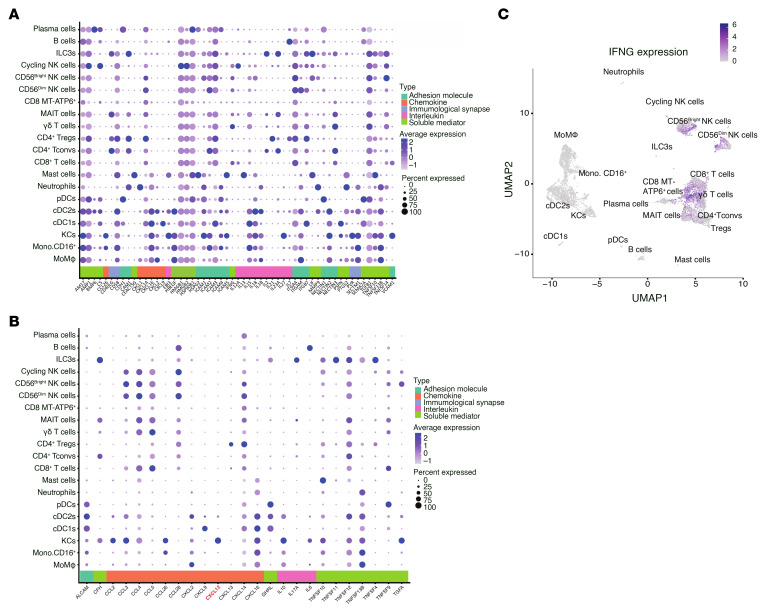
Ligand expression and *IFNG* interactions in intrahepatic CD45^+^ cells. (**A**) Dot plot of selected ligands in intrahepatic CD45^+^ populations, stratified by biological function. (**B**) Dot plot of ligands from intrahepatic CD45^+^ populations interacting with IFNG^+^ NK cells, stratified by function. (**C**) Feature plot of *IFNG* expression on intrahepatic CD45^+^ cells.

The identification of an *IFNG*-associated signature within intrahepatic CD56^bright^ and CD56^dim^ cells suggests that the activation of this immune pattern might be elicited by common signals in CRLM patients. We thus investigated how intrahepatic CD56^bright^ and CD56^dim^ NK cell subsets interact with different components of the CRLM immune microenvironment. We used NicheNet, an algorithm that infers ligand-target activities on the basis of their expressed genes (47). First, scRNA-Seq analysis of intrahepatic CD45^+^ cells of the same patients was used to annotate leukocyte clusters (Figure 7A) (48). We set the three NK cell clusters emerging from CD45^+^ cell analysis (CD56^bright^, CD56^dim^, and cycling NK_LR_ cells) as “receiver” populations, focusing our analysis on the top-scored predicted NK ligands expressed in the “sender” myeloid and lymphoid cells (Figure 7B). Intrahepatic CD56^bright^ and CD56^dim^ NK cells shared most of their ligands upon interaction with either lymphoid or myeloid compartments. In contrast, cycling intrahepatic NK cells established specific ligand-target interactions. Ligands interacting with intrahepatic CD56^bright^ and CD56^dim^ NK cells, including *IFNG*, *TGFB1*, *CD40LG*, and the cytokines *IL2*, *IL21*, and *IL23A*, were largely expressed in lymphoid cells. *IL18*, *IL1B*, *IL27*, *PTGS2*, and *IL15* were mainly expressed by myeloid senders (Figure 7C). Predicted ligands were involved in intrahepatic NK cell–mediated killing (e.g., *PRF1*, *GZM*s, *FASLG*), cytokine secretion (e.g., *CCL3–5*, *XCL1*, *IRF*s), activation and maturation (e.g., *JUN*, *NFKBIA*, *MAFF*, *TBX21*, *NCR3*), and cell migration (e.g., *ICAM1–2*, *ITGAM*). These results suggested that intrahepatic NK cell cytotoxicity was regulated by *IL2* and *TGFB1* expressed by CD4^+^ T cells, type 3 innate lymphoid cells (ILC3s), and γδ T cells (Figure 8A). In addition, we found *IL18* as a key *IFNG*-upstream ligand for both intrahepatic CD56^bright^ and CD56^dim^ NK cells in CRLM patients. Kupffer cells (KCs) and type 2 conventional dendritic cells (cDC2s) were identified as a major source of *IL18*. Focusing on *IFNG-*expressing cells, we found that intrahepatic NK cell ligands were broadly expressed among lymphocytes and myeloid cells (Figure 8, B and C). In contrast, *CXCL12*, the ligand for CXCR4 receptor, was uniquely expressed by KCs, suggesting they regulate the CXCL12/CXCR4 axis in the CRLM milieu.

### Checkpoint landscape defines NK cell identity in CRLM.

Using the scRNA-Seq dataset of CD45^+^ cells in CRLMs (Supplemental Figure 5), we conducted a parallel evaluation of NK cells and other lymphocyte types. Peculiarly, most lymphocyte clusters showed high expression of tissue-affinity genes such as *EOMES*, *CXCR6*, and *CD69*, especially mucosal-associated invariant T (MAIT), CD8^+^, and γδ T cells. In addition, CD3^+^ T cells displayed effector function molecules described in intrahepatic CD56^bright^ NK cells, but not in CD56^dim^ clusters. For instance, γδ T cells and ILC3s are characterized by high levels of *XCL1* and *XCL2*. Furthermore, *GZMK* has been detected in several clusters but not in CD56^dim^ NK cells. Notably, MAIT, CD8^+^, and γδ T cells markedly contribute to TNF and IFN-γ production, while CD56^dim^ NK cells are the predominant cytotoxic population, as suggested by the combination of *GZMB*, *GNLY*, and *PRF1* levels. The evaluation of TFs has further revealed that *TOX2*, a strong terminal maturation/exhaustion marker in lymphocytes, is unexpectedly characteristic of CD56^bright^ NK cells and ILC3s.

IC analysis revealed a shared expression of regulatory molecules such as *CD96*, *SIGIRR*, and *CXCR4*, across distinct immune lymphocyte types, suggesting their common involvement in orchestrating immune regulation within CRLM. However, combinatorial IC pattern analysis uncovered cell type–specific IC signatures. For instance, NK_LR_ cells differed markedly from T cells by lacking expression of *LAG3*, *PDCD1*, and *KLRG1*. In contrast, NK_LR_ cell subsets displayed elevated expression of *KLRC1*, *ENTPD1*, *HAVCR2*, and *CD160*, with expression levels varying between CD56^dim^ and CD56^bright^ NK_LR_ cells. These data point to a finely tuned, subset-specific regulatory landscape that defines NK cell identity and function within the CRLM microenvironment. These distinct checkpoint architectures may pave the way for subset-tailored immunotherapeutic strategies aimed at reinvigorating NK cell–mediated antitumor activity in CRLM.

### Dissection of PB NK cell heterogeneity in CRLM.

**Figure 9:**
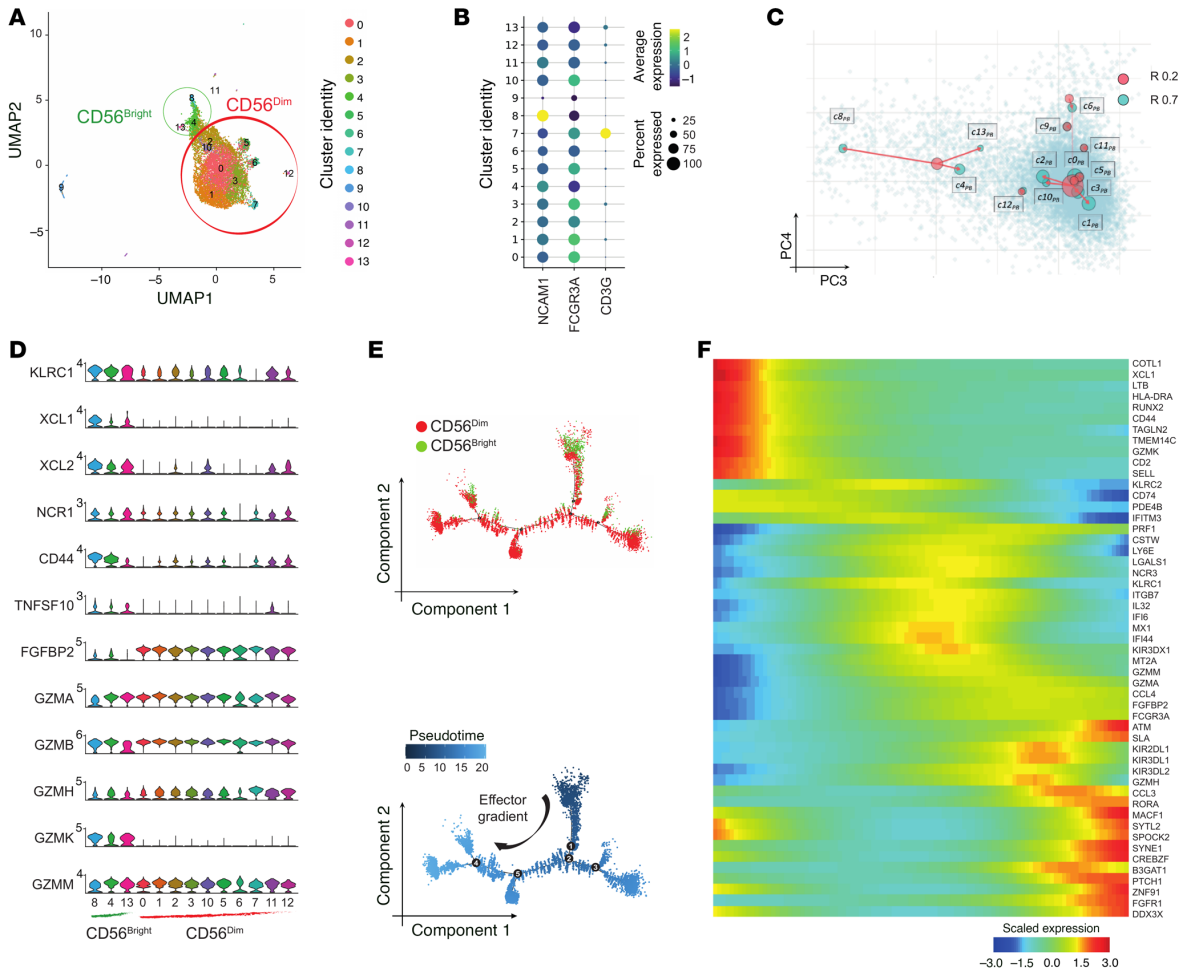
Single-cell RNA-Seq analysis of PB NK cells in CRLM patients. (**A**) UMAP projection of integrated PB NK (NK_PB_) cells (*n* = 12,820) from 3 patients, showing CD56^bright^ (green) and CD56^dim^ (red) clusters. (**B**) Dot plot of *NCAM1*, *FCGR3A*, and *CD3G* expression. (**C**) Overlaying clustering tree of PC3 and PC4 showing CD56^bright^ and CD56^dim^ cells at resolutions 0.2 (pink) and 0.7 (blue). (**D**) Violin plots of selected genes characteristic of CD56^bright^ (green) and CD56^dim^ (red). (**E**) Pseudotime trajectory of NK_PB_ cells colored by cell family (top) or pseudotime value (bottom). (**F**) Heatmap of selected genes along pseudotime.

**Figure 10:**
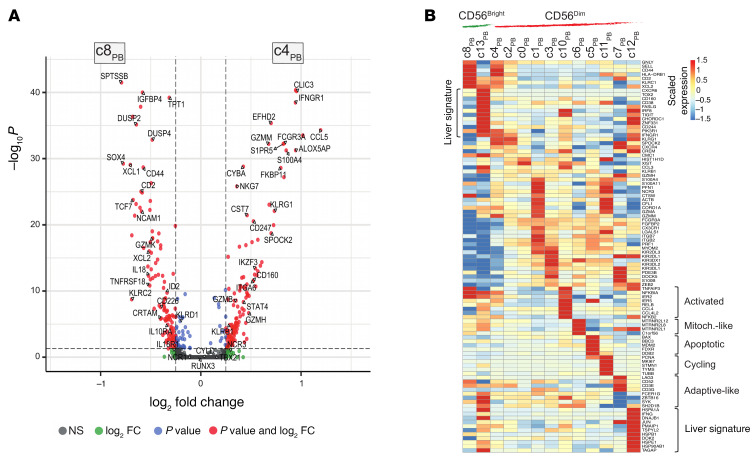
Differential gene expression in NK_PB_ cell clusters. (**A**) Volcano plot of DEGs for CD56^bright^ c8_PB_ versus c4_PB_. (**B**) Heatmap of selected DEGs from CD56^bright^ c8_PB_ versus c13_PB_, and CD56^dim^ c1–7_PB_ and c10–12_PB_ clusters. Expression values are zero-centered and scaled per gene.

In parallel, we performed scRNA-Seq analysis of NK cells isolated from matched PB samples. Based on transcriptomic profiles, a total of 12,820 PB NK (NK_PB_) cells were projected by UMAP. The unsupervised analysis identified 14 cell clusters (c0–13_PB_) with a similar relative frequency among each donor (Figure 9A and Supplemental Figure 6A). The lack of *FCGR3A* and *NCAM1* expression together with a myeloid cell signature prompted us to exclude c9_PB_ from further analysis (Figure 9B and Supplemental Figure 6B). Two main CD56^bright^ and CD56^dim^ phenotypes were annotated by hierarchical clustering and by transcriptional profiles (Figure 9, C and D, Supplemental Figure 6, C and D, and Supplemental Table 2) (33, 34). CD56^bright^ NK_PB_ cells included 3 clusters (c8_PB_, c4_PB_, c13_PB_) that represented a minority (9.6%) of total NK_PB_ cells. CD56^dim^ NK_PB_ cells were grouped into 4 major (c0–3_PB_) and 5 minor (c5–7_PB_, c11–12_PB_) clusters. In contrast to their intrahepatic counterparts, CD56^bright^ and CD56^dim^ NK_PB_ cells showed interrelated differentiation states with continuous progression. The pseudotime trajectory began with the CD56^bright^ c8_PB_ cluster expressing immature NK cell markers such as *COTL1*, *SELL*, *CD44*, *CD2*, *XCL1*, *XCL2*, and *GZMK* (Figure 9, E and F, and Supplemental Figure 7, A–C). Transitional states between CD56^bright^ and CD56^dim^ phenotypes were observed in clusters c4_PB_ and c2_PB_, as suggested by increased effector potential (e.g., *GZM*s, *FCGR3A*, *CTSW*, *KLRB1*, *NKG7*, *TBX21*, *NCR3*) and lower expression of CD56^bright^ markers (e.g., *XCL1*, *XCL2*, *CD44*, *GZMK*, *NCR1*) in comparison with c8_PB_ (Figure 9, D and F, and Figure 10A). Likewise, NK cells in c2_PB_ showed a less mature phenotype in comparison with the other CD56^dim^ clusters (Figure 10B). We further detected increased expression of *CX3CR1* in c1_PB_ indicating preferential migration of mature CD56^dim^ cells to the CRLM microenvironment (Figure 1D).

CD56^bright^ (c13_PB_) and CD56^dim^ (c5–7_PB_ and c10–12_PB_) cells showed tumor-related transcriptomic profiles (Figure 10B). In particular, c13_PB_ showed tissue residency and effector genes previously identified in intrahepatic CD56^bright^ cells (e.g., *CXCR6*, *FASLG*, *IRF8*, *TIGIT*, *CD160*, *TOX2*, *CHORDC1*), thus suggesting their egression from the liver to the blood. CD56^dim^ cells in c6_PB_ expressed a family of mitochondrial genes (e.g., *MTRNR2L8*/*12*) (49, 50). CD56^dim^ NK_PB_ cells in c5_PB_ and c11_PB_ were characterized by an apoptotic (e.g., *BAX*, *BBC3*, *MDM2*) and a proliferating (e.g., *MIK67*, *PCNA*, *STMN1*) phenotype, respectively. CD56^dim^ c12_PB_ cells, similarly to c13_PB_, mirrored a liver signature (i.e., *IFNG*, *DNAJB1*, *HSPA1A*, *HSPB1*). Finally, CD56^dim^ c10_PB_ showed an activated phenotype (e.g., *IRF8*, *IER5*, *IER2*, *NFKBIA*, *RELB*) and c7_PB_ an adaptive-like profile as indicated by higher expression of *CD3E*, *CD3G*, *CD52*, and *LAG3* and lower expression of *FCER1G* (FcεRγ), *ZBTB16*, *SYK*, *SH2D1B* (EAT2), and *KLRB1* (43, 51).

### Multiparametric flow cytometry profiling of NK cells in CRLM patients.

**Figure 11:**
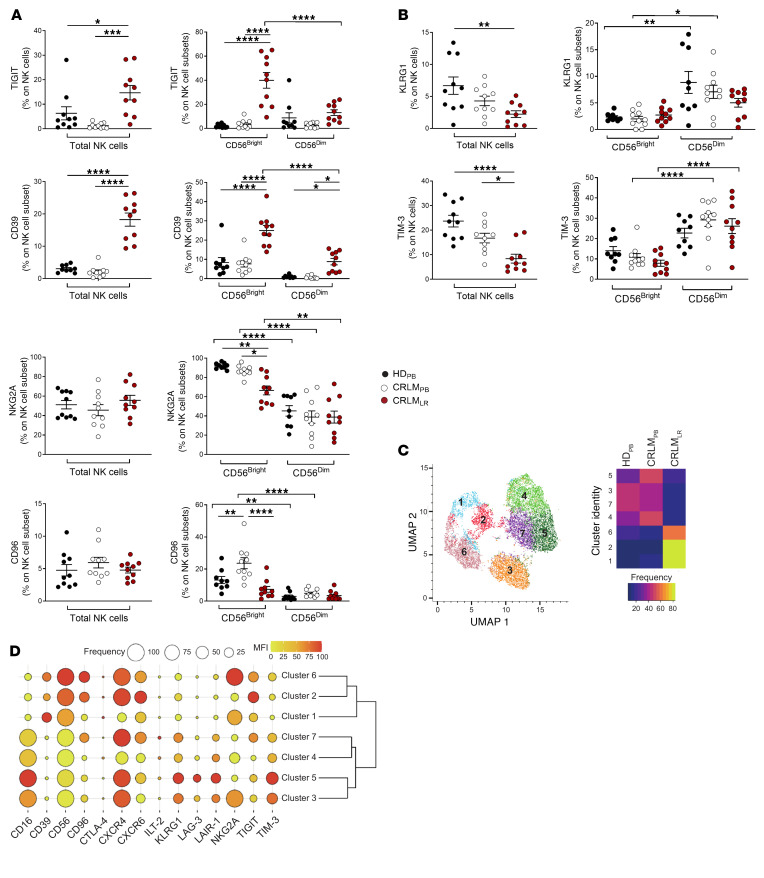
Flow cytometry analysis of blood and tissue NK cells in CRLM patients. (**A** and **B**) Flow cytometry of immune checkpoint expression in NK cells from HD_PB_, CRLM_PB_, and CRLM_LR_. Data are shown as mean ± SEM. *P ≤ 0.05; **P ≤ 0.01; ***P ≤ 0.001; ****P ≤ 0.0001. One-way ANOVA (left) or 2-way ANOVA (right); *n* = 10 per group. (**C**) UMAP projection showing relative abundance of NK cell clusters in HD_PB_, CRLM_PB_, and CRLM_LR_. (**D**) Dot plot of marker frequency (percent) and MFI across NK cell clusters.

scRNA-Seq analysis highlighted a specific IC pattern in intrahepatic NK cells depending on tissue residency, effector stage, response to CRLM, and likely egress of intrahepatic NK cells to bloodstream. We next proceeded to validate the NK cell expression of ICs by multiparametric flow cytometry of liver samples and PB isolated from CRLM patients and compared with PB of healthy donors (HDs). We confirmed that intrahepatic NK cells expressed high levels of TIGIT, CD39, NKG2A, and CD96, although with high heterogeneity among patients. Notably, TIGIT and CD39 were mainly enriched on CD56^bright^ NK_LR_ cells from CRLM patients (Figure 11A), and in line with the transcriptional profiles, the expression of these two ICs was higher on CD56^bright^ NK_LR_ cells compared with their PB counterparts. In contrast, CD56^bright^ NK_PB_ cells presented higher expression of NKG2A and CD96 compared with CD56^bright^ NK_LR_ cells. TIM-3 and KLRG1 were preferentially expressed by both intrahepatic and circulating CD56^dim^ NK cells (Figure 11B). The coexpression of different ICs was evaluated by multiparametric PhenoGraph algorithms (Figure 11, C and D), which generated 3 clusters of CD56^bright^ NK cells (c1–2, c6) and 4 clusters of CD56^dim^ NK cells (c3–5, c7). These analyses confirmed the differential and peculiar NK cell expression patterns of ICs in CRLM. Indeed, CD56^bright^ NK_LR_ cells in c1 and c2 showed higher expression of TIGIT and CD39, each mutually excluded (Figure 11D). High expression of NKG2A was found in c6 embedded in both circulating and intrahepatic CD56^bright^ cells. The 2 CD56^dim^ NK_PB_ cell clusters c4–5, highly enriched in CRLM, were also characterized by high expression of TIM-3, LAIR-1, and KLRG1 (Figure 11D).

**Figure 12:**
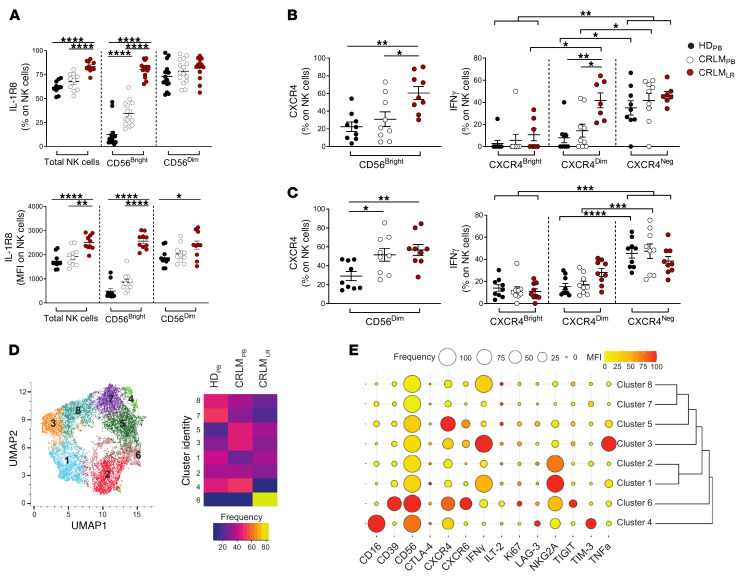
Evaluation of IL-1R8 and CXCR4^+^ NK cell profiling in CRLM. (**A**) IL-1R8 expression in NK cells from HD_PB_, CRLM_PB_, and CRLM_LR_. Top: Relative frequency (percent) in total NK, CD56^bright^, and CD56^dim^ subsets. Bottom: The MFI in the same subsets. (**B** and **C**) CXCR4 (left) and IFN-γ (right) expression in CD56^bright^ (**B**) and CD56^dim^ (**C**) NK cells from HD_PB_, CRLM_PB_, and CRLM_LR_, stratified by CXCR4 levels. (**A**–**C**) Data are shown as mean ± SEM. *P ≤ 0.05; **P ≤ 0.01; ***P ≤ 0.001; ****P ≤ 0.0001. One-way ANOVA (**A**; **B** and **C**, left) or 2-way ANOVA (**B** and **C**, right). (**A**) *n* = 15 (HD_PB_, CRLM_PB_), *n* = 14 (CRLM_LR_); (**B** and **C**) *n* = 9. (**D**) UMAP projection showing NK cell cluster distribution across HD_PB_, CRLM_PB_, and CRLM_LR_. (**E**) Dot plot of marker frequency (percent) and MFI in NK cell clusters.

We then evaluated the expression of CXCR4 and IL-1R8 (*SIGIRR*). IL-1R8 is an IC whose expression is upregulated following NK cell maturation (31, 52). Accordingly, CD56^dim^ NK_PB_ cells expressed high levels of this molecule (Figure 12A). Surprisingly, we also observed an increased amount of IL-1R8 on CD56^bright^ NK_PB_ cells from CRLM patients compared with HDs. In line with our transcriptional scRNA-Seq analysis, both intrahepatic CD56^bright^ and CD56^dim^ NK cells showed the highest levels of IL-1R8 in CRLM (Figure 12A). Similarly, the highest percentages of CXCR4^+^ cells were found in both intrahepatic CD56^bright^ and CD56^dim^ NK cells (Figure 12, B and C). We then investigated the association between the levels of CXCR4 expression and IFN-γ production. To this end, CD56^bright^ and CD56^dim^ cells were stratified on the basis of the different intensity of CXCR4 expression. First, our results confirmed that CD56^bright^ NK_LR_ cells produced IFN-γ (Figure 12B). Then, CXCR4^dim/neg^ showed higher IFN-γ levels compared with CXCR4^bright^ in CRLM from both intrahepatic CD56^bright^ and CD56^dim^ NK cells (Figure 12, B and C). These results were also confirmed by multiparametric PhenoGraph analysis (Figure 12, D and E). CXCR4 was associated with the liver residency marker CXCR6, TIGIT, and CD39 in cluster 6 (Figure 12E). Finally, CXCR4^+^CXCR6^+^ NK cells (cluster 5) were observed only in the PB of patients with CRLM, not in HDs.

### CXCR4 and IL-1R8 blockade enhances NK cell effector function in CRLM.

**Figure 13:**
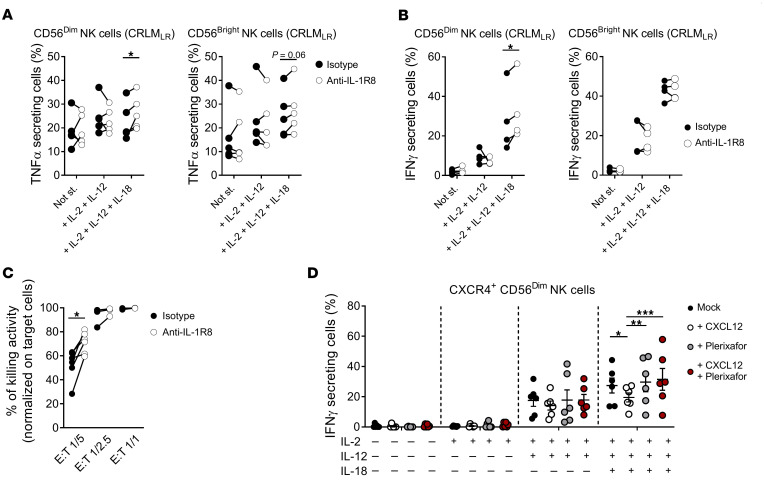
Targeting IL-1R8 and CXCR4 in NK cells from CRLM. (**A** and **B**) TNF-α (**A**) and IFN-γ (**B**) expression in liver CD56^dim^ and CD56^bright^ NK cells from CRLM_LR_ patients stimulated with anti–IL-1R8 mAb or isotype control. (**C**) Cytokine-stimulated PB NK cells (*n* = 6, HDs) show increased cytotoxicity upon IL-1R8 blockade in HT-29 killing assay at indicated effector-to-target (E:T) ratios (1:5, 1:2.5, 1:1). (**D**) IFN-γ expression in CXCR4^+^CD56^dim^ liver NK cells from CRLM patients stimulated with CXCL12 and/or plerixafor. (**A**–**D**) Data are shown as mean ± SEM. *P ≤ 0.05; **P ≤ 0.01; ***P ≤ 0.001. Statistical analysis was performed using paired 2 tailed parametric *t* test (**A** and **B**), paired 2 tailed non-parametric test (**C**), and 2-way ANOVA (**D**). Sample size: *n* = 3–6 for **A–C**; *n* = 4–6 for **D**.

We next investigated the functional relevance of IL-1R8 and CXCR4 in NK_LR_ and NK_PB_ cells. To properly activate NK cells, we stimulated isolated CRLM lymphocytes and PBMCs with the cytokines IL-2, IL-12, and IL-18 (53). Targeting IL-1R8 with a blocking mAb increased the effector functions of intrahepatic CD56^dim^ NK cells, as indicated by the higher intracellular levels of TNF-α and IFN-γ upon stimulation with IL-2, IL-12, and IL-18, in 5 of 6 and 4 of 6 CRLM patients, respectively (Figure 13, A and B, and Supplemental Figure 8, A and B). These results indicate that despite NK cell subsets expressing comparable levels of IL-1R8, they exhibit distinct functional properties upon IL-1R8 blockade in this experimental condition. In addition, blocking IL-1R8 would result in unleashing specific functional features, depending on the effector potential, transcriptional profile, and repertoire of activating and inhibitory molecules of NK cell subsets (Figure 13C).

Finally, we investigated the role of CXCR4 in intrahepatic NK cells by stimulating them with CXCL12 and/or with the CXCR4 antagonist plerixafor. Intrahepatic CD56^dim^ NK cells (but not CD56^bright^) showed a significantly decreased production of IFN-γ upon stimulation with CXCL12 when compared with controls (Figure 13D). This impairment was restored by plerixafor, which counteracted CXCL12-mediated inhibition of IFN-γ production of intrahepatic CD56^dim^ NK cells (Figure 13D). These results indicate that the effector functions of CXCR4^+^CD56^dim^ NK cells infiltrating CRLM are tuned by the CXCR4-ligand interactions.

## Discussion

Heterogeneity of tumor-infiltrating immune cells represents a major challenge in achieving clinical benefits from immunotherapy in CRLM. Several lines of evidence demonstrated that liver tumor microenvironment (TME) actively participates in the establishment and progression of metastases (54, 55). The present study investigates the complexity of matched intrahepatic and circulating NK cells at single-cell level in patients undergoing surgical resections of CRLM lesions after the administration of na-CHT treatment.

CD56^bright^ NK cells are considered precursors of mature CD56^dim^ NK cells (56). In line with other studies (33, 57), we confirmed a developmental trajectory from CD56^bright^ to CD56^dim^ and the existence of transitional differentiation stages in circulating NK cells. The expression of *CX3CR1*, a marker associated with the tissue recruitment of mature NK cells (40–42), strongly suggests that circulating CD56^dim^ NK cells infiltrate the CRLM and work in synergy with NK_LR_ cells. Conversely, we show here that intrahepatic CD56^bright^ and CD56^dim^ cells in CRLM have different developmental origin, end-terminal differentiation programs, and conserved effector gene signatures. The CD56^bright^ cell subsets represented the vast majority of NK_LR_ cells and expressed liver-residency genes (*CXCR6*, *CCR5*, *EOMES*, and *CD69*). Moreover, they are characterized by TFs such as *IRF8*, *EOMES*, and *TOX2* that are required for functional maturation of human NK cells (35–37, 39). For instance, *TOX2* upregulation is directly involved in maturation and cytotoxicity in human NK cells (35). In agreement with TF profiling, intrahepatic CD56^bright^ NK cells from CRLM patients expressed high levels of effector function molecules (e.g., *GZMK*, *GZMA*, *FASL*) (43, 58, 59). Hence, in contrast with their circulating counterparts, CD56^bright^ NK_LR_ cells can be considered mature and terminally differentiated lymphocytes. Moreover, tissue-resident NK cells expressed cluster markers of CD56^dim^ cells from PB (e.g. *CCL4*, *CCL5*, *NKG7*, and *IL32*) (33), reflecting the unique ontogeny and activation state in the CRLM contexture. Single-cell transcriptomic profiling revealed a strong similarity between NK cells isolated from PT and IM tissues, suggesting that NK cells undergo early commitment and functional adaptation independently of direct tumor–NK cell interactions. This highlights the potential role of soluble factors in modulating NK cell differentiation and antitumor activity. In contrast, myeloid cell clusters from the same samples displayed distinct distributions between the two regions, indicating a more localized TME-dependent adaptation (48).

We recently identified a subset of tumor-infiltrating γδ T lymphocytes that recirculate in PB of CRLM patients, where they retained a transcriptional signature and γδ T cell receptor repertoire resembling their liver origin and positively correlated with longer overall survival in CRLM patients (60). Along this line, we detected rare circulating NK cells showing liver signatures, suggesting that intrahepatic NK cells leave CRLM and recirculate into the bloodstream, where they retain distinctive transcriptional profiles, but further studies are needed to evaluate their association with the clinical outcome.

Clinical trials are investigating the outcome of CRLM patients treated with immune checkpoint inhibitors (ICIs) (61, 62). Hence, the characterization of IC pattern among tumor-infiltrating NK cells is key to optimize immunotherapies. The present study depicts specific expression patterns of ICs by CD56^bright^ and CD56^dim^ NK cells in CRLM patients. CD56^bright^ cells expressed high levels of NKG2A, which is currently under assessment in several clinical trials (63, 64). Higher levels of TIM-3, KLRG1, and KIRs were found in CD56^dim^ NK cells, whose targeting is under investigation (62). In addition, we identified TIGIT, CD96, and CD39 as ICs specifically expressed by CD56^bright^ NK_LR_ cells. Notably, TIGIT and CD39 were shown to be upregulated in PB NK cells from patients with acute myeloid leukemia and in esophageal squamous cell carcinoma, respectively (65, 66), suggesting specific IC regulation mechanisms that depend on the tumor type.

The clinical relevance of NK cell–mediated immune surveillance in liver metastatic diseases had been extensively reported (22, 29–31). Here, we identified an *IFNG^high^* signature associated with both intrahepatic CD56^dim^ and CD56^bright^ NK cells from CRLM. IFN-γ is the critical effector factor determining the success of immunotherapy and represents an important prediction marker for the clinical response to ICIs (30). Indeed, high *IFNG* and *IFNG*-related gene signatures in patients with different primary and metastatic tumors (i.e., melanoma, head and neck squamous cell carcinoma, gastric cancer, lung cancer) were associated with effective ICI treatments (67–70). Recent studies showed that IFN-γ–secreting lymphocytes modify the TME (71, 72). IFN-γ administration has been proposed to improve the efficacy of PD-1 blockade therapy in pancreatic cancer (73) and is under clinical evaluation (e.g., ClinicalTrials.gov NCT02614456, NCT03063632). The *IFNG^high^* signature identified in our study is also enriched in several activation molecules (*JUN*, *GIMAP4*, *DUSP*), suggesting the antitumor potentiality of liver NK cells. On the other hand, the signature is enriched also in stress-induced molecules (*HSP*s), which have been associated with a dysfunctional state (57).

In this study, we show that IL-1R8 is ubiquitously and highly expressed by all human intrahepatic NK cells in CRLM. We have previously shown that IL-1R8 acts as an IC dampening IL-18–dependent NK cell activation in mouse models of primary and metastatic liver cancer (31). Here we show that myeloid-derived IL-18 emerges as a pivotal upstream regulator in CRLMs. We also provide evidence that an anti–IL-1R8 blocking mAb increases the functional activation of cytokine-stimulated intrahepatic NK cells, although with variability among patients, supporting the hypothesis that IL-1R8 could be a target for immunotherapy. In preclinical models, IL-1R8 deficiency increased susceptibility to colitis-associated chemical carcinogenesis (74), while enhancing protection from primary liver carcinogenesis and CRLM (23). These opposite effects are likely explained by the fact that IL-1R8 is expressed in colon epithelial cells as well as NK cells. IL-1R8 systemic blockade has the risks associated with exacerbated inflammatory reactions, in parallel with unleashed antitumor activity of NK_LR_ cells. The development of IL-1R8 as IC in cancer would imply NK-directed targeting, for instance through genetic silencing.

In addition, we observed that both intrahepatic CD56^dim^ and CD56^bright^ NK cell subsets displaying *IFNG^high^* signatures were further characterized by lower expression of CXCR4, a chemokine receptor involved in leukocyte bone marrow retention and in NK cell maturation (75, 76). Recent studies extended its biological significance, showing that hepatic stellate cells and fibroblasts secrete CXCL12 (11), which, in turn, hampered NK cell–mediated resistance against liver metastasis by engaging CXCR4 (44). We observed that among CD45^+^ liver cells, KCs expressed high levels of CXCL12, and that the stimulation of intrahepatic CD56^dim^ NK cells with CXCL12 limited their functional activity, which was rescued by the CXCR4 antagonist plerixafor.

In summary, the present study discloses the origin, heterogeneity, and effector functions of matched circulating and intrahepatic NK cells from CRLM patients by using single-cell technologies and unbiased analytic approaches, shedding light on an abundant *IFNG^high^* resident CD56^bright^ NK cell population with an antitumor signature, and providing two possible therapeutic targets (IL-1R8 and CXCR4) to complement current immunotherapy of CRLM.

## Methods

### Sex as a biological variable

Our study examined male and female individuals, and sex was not considered as a key biological variable.

### Study design

The study investigated the role of NK cells in patients with histologically proven CRLM older than 18 years of age who underwent hepatectomy at IRCCS (Istituto di Ricovero e Cura a Carattere Scientifico [Scientific Institute for Research, Hospitalization and Healthcare]) Humanitas Research Hospital, mostly represented by patients with synchronous clinical profile treated with standard combination bevacizumab/cetuximab na-CHT 2–4 weeks before surgery. The preoperative work-up consisted of liver-specific MRI and total-body contrast-enhanced CT, performed a maximum of 30 days before surgery. Matched specimens from the invasive margin (IM) and peritumor (PT) area as well as from PBMCs of 3 patients with CRLM were processed for scRNA-Seq. Specifically, scRNA-Seq analysis was performed on flow cytometry–sorted NK cells (Lin^–^CD56^+^) excluding CD3^+^ lymphocytes and myeloid and B cells (altogether referred to as Lin^+^), and on total CD45^+^ leukocytes that were freshly isolated from the matched samples of 3 representative patients (2 male and 1 female) as well as on matched PBMCs.

### Tissue processing and cell preparations

Peripheral blood mononuclear cells (PBMCs) were isolated from buffy coats of healthy donors (HDs) or from PB of CRLM patients by Lympholyte Cell Separation density gradient solution (Cedarlane Laboratories) according to the manufacturer’s instructions. Liver tissues were dissociated by enzymatic digestion in Hanks balanced salt solution with Ca^2+^ and Mg^2+^ (HBSS^+/+^, Euroclone SpA) with 2 mg/mL of collagenase D (Sigma-Aldrich), 50 μg/mL DNase I (Sigma-Aldrich), 2% fetal bovine serum (FBS) (Sigma-Aldrich), and 10 mmol/L HEPES (Lonza) in gentleMACS Dissociator (Miltenyi) for 35 minutes at 37°C/5% CO_2_. Cells were then filtered through a 70 μm cell strainer (Corning) and washed with HBSS without Ca^2+^ and Mg^2+^ (Euroclone SpA). Lymphocytes were separated by 70%/30% discontinuous Percoll gradient (Cytiva) and frozen in FBS (Lonza) with 10% of dimethylsulfoxide (Lonza) before being stored in liquid nitrogen for further analysis.

### Flow cytometry analysis

For multiparametric flow cytometry analysis, cells were stained with Zombie Aqua fixable viability kit (BioLegend) for live/dead discrimination. Then, cells were washed with HBSS^–/–^ with 2% of FBS (FACS buffer) and incubated with the mix of mAbs for 20 minutes in the dark at room temperature, washed again with FACS buffer, and fixed in 1% of paraformaldehyde (Santa Cruz Biotechnology). All reagents and mAbs are listed in Supplemental Table 1.

The intracellular concentrations of IFN-γ and TNF-α were measured by flow cytometry using anti–human IFN-γ mAb (clone B27; BioLegend, catalog 506518) and anti–human TNF-α mAb (clone Mab11; BioLegend, catalog 506518) and were evaluated after stimulation of PBMCs and liver-infiltrating lymphocytes with 1 μg/mL of GolgiPlug protein transport inhibitor (BD Biosciences). After 4 hours at 37°C/5% CO_2_, cells were collected and washed with HBSS^–/–^, and cellular membrane staining was done as described above. Subsequently, intracellular staining was performed with a Cytofix/Cytoperm kit (BD Biosciences) according to the manufacturer’s instructions.

All samples were acquired by a FACSymphony A5 flow cytometer (BD Biosciences). Flow cytometry data were compensated using single-stained controls with BD Compbeads (BD Biosciences) conjugated to the specific fluorescent mAb. For accurate flow cytometry practice, all mAbs were previously titrated (77). All flow cytometry data, including the dimensionality reduction method with uniform manifold approximation and projection (UMAP) algorithm and unsupervised clustering with PhenoGraph algorithm, were analyzed by FlowJo software version 10.8.1 (FlowJo LLC).

### Library preparation and sample sequencing

NK cells were sorted by flow cytometry (BD FACSAria III Cell Sorter) as lineage negative and CD56^+^, isolated from PB and liver of 3 CRLM patients, and were subjected to scRNA-Seq analysis. NK cell purity after FACS sorting was 97% or greater. In collaboration with the Genomic Unit of Humanitas Research Hospital, scRNA-Seq libraries were prepared using the Single Cell 3′ Library and Gel Bead Kits (10x Genomics). According to the established protocol, approximately 10,000 cells for each sample were loaded into the Chromium controller (10x Genomics) to generate the Gel Beads-in-Emulsion (GEMs). The libraries were sequenced using the Illumina NextSeq500 platform.

#### Processing scRNA-Seq data.

3′-based sequencing data were aligned and quantified using the Cell Ranger Single-Cell Software Suite (v3.0.2, 10x Genomics) to the GRCh38 (refdata-gex-GRCh38-2020-A) human reference genome. Subsequent analyses were performed using the R package Seurat (78) (v3.1.1; R v3.6.1). Cells were filtered based on quality metrics to exclude low-quality, potentially dying cells, or doublets. Specifically, cells expressing fewer than 200 genes or more than 6,400 genes, with fewer than 365 unique molecular identifiers (UMIs) or more than 43,000 UMIs, or with mitochondrial gene content exceeding 20% were removed. PB NK cells and integrated PT and IM NK cells from 3 CRLM patients were first analyzed separately. Specifically, the *FindIntegrationAnchors***()** and *IntegrateData()* functions were used to combine tissue NK cells of 3 CRLM patients as well as NK cells from PB. Principal component analysis (PCA) was performed with the *RunPCA()* function. To reduce the dimensionality of the dataset, the *RunUMAP()* function was used on the pre-computed principal components. The clusters were identified with the *FindClusters()* function based on pre-computed principal components and visualized by UMAP plot. Eighteen and fourteen clusters were identified at resolution 0.7 for tissue and PB, respectively. PT and PB NK cells were then integrated, and 18 clusters were identified at resolution parameter 0.8. For each integrated dataset, NK cell clusters were annotated based on the average expression of canonical NK cell markers. The default Wilcoxon’s rank-sum test was used by running of the *FindAllMarkers()* function to find differentially expressed markers in each cluster. The upregulated or downregulated genes with |log_2_(fold change)| greater than 0.25 and *P* value less than 0.05 were considered significant DEGs.

### Gene Ontology enrichment analysis

Gene Ontology (GO) (biological process) enrichment analysis was performed by the R package clusterProfiler (79). The GO pathways of each cell type were enriched using DEGs with FDR-adjusted *P* value ≤ 0.05 and log(fold change) > 0.25. Only enriched GO terms with *q* value ≤ 0.01 were selected as significant and visualized by the R package ggplot2. Dots were sized by adjusted *P* value and colored by EnrichFold values defined as the ratio between BgRatio and GeneRatio terms from enrichment analysis. *IFNG^high^* signature was defined by selection of shared DEGs with FDR-adjusted *P* value ≤ 0.05 and log(fold change) > 0.25, from the comparison of c5_LR_ versus c0–1–2_LR_ and c6_LR_ versus c3–4_LR_.

### Pseudotime ordering

#### Monocle2.

Pseudotime trajectory analysis was performed separately on tissue and PB by Monocle (v2.8.0) (80). Cells were ordered along the trajectory by the *dpFeature* unsupervised procedure. Only genes expressed in at least 5% of all the cells were selected, and the top 1,000 highly variable genes (HVGs) were used as the ordering genes. For tissue trajectory, c8_LR_ was selected as the root node of the graph, while for blood trajectory, c8_PB_ was selected as the root node. The *DifferentialGeneTest()* function was used to test for a significant correlation between gene expression and pseudotime. A gene was defined as significantly associated with pseudotime if its estimated *q* value was lower than 0.01. Significantly associated genes were visualized by the *plot_pseudotime_heatmap()* function.

#### Slingshot.

Pseudotime trajectory inference was performed using the Slingshot algorithm (v2.12.0) (81) in R (v4.4.0) and Seurat (v4.4.0). Only cells belonging to NK cell clusters from tissue and blood objects were selected for downstream analysis. On the selected cells, a new UMAP was computed to include only NK cells.

Subsequently, the 2 new datasets were independently converted into a SingleCellExperiment object, using the log-normalized expression matrix and cluster identities. UMAP embeddings were imported into the SingleCellExperiment object using the *reducedDim()* function. Slingshot was run using the *slingshot()* function, specifying the original Seurat clusters as lineage labels and the UMAP embedding as the reduced dimensionality space and setting cluster 8 as the starting point for both the tissue and blood. The distance method was set to “simple,” and the omega parameter was enabled to allow adaptive weighting of constraints.

Pseudotime values were extracted using the *slingPseudotime()* function, and shared pseudotime was calculated as the mean pseudotime across the inferred lineages.

### Public healthy liver scRNA-Seq databases

Raw counts of healthy liver samples (*n* = 5) were downloaded from the NCBI’s Gene Expression Omnibus (GEO) database (GSE136103) (45) and processed in Python (v3.9.7) using Scanpy (v1.9.3) (82). Each sample underwent independent quality control: cells with fewer than 500 or more than 4,000 expressed genes, fewer than 950 or more than 12,000 UMIs, greater than 10% mitochondrial content, or less than 0.05% ribosomal content were filtered out. Doublets were removed using Scrublet (v0.2.3) (83). Samples were merged into a single AnnData object (v0.10.4) (84) and log-normalized (scale factor 10,000). HVGs (*n* = 4,000) were identified using Seurat_v3 dispersion-based methods, followed by PCA. Batch correction and data integration were performed with pyHarmony (v1.0.21) (85) setting “patient_ID” as batch key. Clustering was performed using the Leiden algorithm (86) and visualized via UMAP (87) in the Harmony-corrected space. For NK cell re-clustering, selected cells were extracted, HVGs and PCA were re-computed, and integration was repeated with the same parameters.

### Integration of healthy liver and tumor-associated NK cells

Raw count matrices of tumor-associated NK cells were first exported from Seurat and imported into Python (v3.9.7) for integration with healthy NK cells. Data integration and batch correction were performed using the single-cell Variational Inference (scVI)-tools algorithm (v1.0.0) (88), setting both “sequencing_ID” and “sample_ID” as batch key covariates.

For DEG analysis, the raw count matrix of the integrated dataset was exported from Python and imported into R (v4.4.0) as a Seurat object, generated using the *CreateSeuratObject()* function (Seurat v4.4.0). DEGs were computed using the Seurat *FindMarkers()* function, applying a negative binomial test, setting “sequencing_ID” for batch correction. Significant DEGs were defined as those with an adjusted *P* value less than 0.05 and |log_2_(fold change)| > 0.25.

### NicheNet interaction analysis

The analysis to predict the existing ligand-target interaction between the CD45^+^ population and NK cells was done with NicheNet (v1.0.0, Seurat v3.6). The normalized data obtained after the Seurat analysis were imputed using the ALRA (adaptively thresholded low-rank approximation) function to correct the missed counts, with a predicted rank-*k* approximation of 35. We set as sender cells, which express the ligand genes, each cluster in the CD45^+^ cell population, and as receiver cells 3 different NK cell clusters (CD56^bright^, CD56^dim^, and cycling NK cells). The analysis was restricted to the genes expressed in at least 50% of sender and receiver cell clusters. The cluster gene markers with adjusted *P* value 0.05 and avg_logFC 0.5 from each receiver cell population were used as the target gene set, whereas the background genes were chosen as expressed in all cell populations with a cluster frequency above 50%. After the ligand activity analysis, only the ligands with a positive Pearson’s correlation coefficient were considered. To visualize the ligand-receptor interactions, we used the circlize R package (v0.4.12).

We gathered together ligands related to myeloid or lymphoid compartments and selected the first 100 interactions ranked by their regulatory potential.

### In vitro stimulation

Human NK cells were cultured in RPMI 1640 medium supplemented with 10% FBS, 1% l-glutamine, 1% penicillin/streptomycin with IL-2 (Proleukyn), IL-12 (Miltenyi), and IL-18 (MBL Life Science). The following reagents were used, as specified: an original mouse anti-hSIGIRR antibody (IgG1, κ isotype, low endotoxin, azide free; generated by the University of Rijeka Faculty of Medicine, Center for Proteomics); Ultra-LEAF Purified Mouse IgG1, κ Isotype Control (Ctrl) Antibody (BioLegend); CXCL12 (PeproTech); plerixafor; and Cell Activation Cocktail (eBioscience), IFN-γ and TNF-α production was evaluated after 16 hours of stimulation with IL-2 (10 ng/mL), IL-12 (10 ng/mL), IL-18 (5 ng/mL), anti-hSIGIRR (1 μg/mL), Ultra-LEAF Purified Mouse IgG1, κ Isotype Ctrl Antibody (1 μg/mL), CXCL12 (100 ng/mL), and plerixafor (100 ng/mL) alone or in combination. The intracellular staining was performed using a BD Cytofix/Cytoperm Fixation/Permeabilization Kit following the manufacturer’s instructions. BD GolgiPlug (containing brefeldin) was added 4 hours before intracellular staining.

### Killing assay

For cytotoxicity experiments, PBMCs were isolated from buffy coats by Lympholyte Cell Separation density gradient solution (Cedarlane Laboratories) according to the manufacturer’s instructions. NK cells were purified by negative selection, using EasySep Human NK Cell Enrichment Kit (Stemcell Technologies), and cultured for 6 days in DMEM/F-12–DMEM high-glucose (1:1) supplemented with 10% Human Serum, Type AB (Capricorn Scientific); 1% l-glutamine; 1% penicillin/streptomycin; 1% sodium pyruvate; 1% non-essential amino acids; IL-15 (10 ng/mL; Miltenyi); and IL-2 (100 IU/mL; Proleukyn). On the day of coculture, HT-29 target cells were detached with Trypsin/EDTA solution (Lonza), washed twice in PBS, and labeled with CellTrace CFSE Cell Proliferation Kit (Thermo Fisher Scientific) following the manufacturer’s protocol. Fifty thousand labeled HT-29 cells were seeded in a flat-bottom 96-well plate 4 hours before the coculture. Cytokine-primed NK cells were collected and cocultured with HT-29 cells at different effector-to-target ratios (1:5, 1:2.5, 1:1) upon 30 minutes of preincubation with anti–hIL-1R8 (1 μg/mL) or Ultra-LEAF Purified Mouse IgG1, κ Isotype Ctrl Antibody (1 μg/mL) at 37°C, which was left throughout the duration of the coculture. Cells were stimulated with IL-15 (20 ng/mL; Miltenyi) and IL-18 (50 ng/mL; MBL Life Science) overnight. To quantify viable HT-29 target cells, supernatants were collected and pooled with the remaining adherent HT-29 cells detached using the trypsin. The number was normalized using CountBright Absolute Counting Beads (Thermo Fisher Scientific). Killing activity was calculated as follows: 1 – (residual number of target cells in the presence of NK cells / number of control target cells).

### Statistics

Flow cytometry analysis was performed using GraphPad Prism v9. ANOVA with Tukey’s multiple-comparison test was used to compare multiple groups. The data are presented as median value ± SEM. Statistically significant *P* values are represented in GraphPad style and are summarized with asterisks as follows: *P < 0.05; **P < 0.01; ***P < 0.001; ****P < 0.0001.

### Study approval

The study protocol was in accordance with the ethical guidelines established in the Declaration of Helsinki, and participants gave written informed consent to participate in the study before taking part. All patients were recruited at the Hepatobiliary and General Surgery Department at Humanitas Clinical and Research Center (HCR), Rozzano, Milan, Italy. Liver tissue and PB specimens were collected from CRLM patients in accordance with clinical protocols approved by the Institutional Review Board of HCR Institute (approval 168/18). PB of HDs was obtained in accordance with HCR and with clinical protocols approved by the Institutional Review Board of Desio Hospital, Milan, Italy.

### Data availability

All data relevant to the study are included in the article or its supplemental information. The filtered scRNA-Seq gene expression counts generated in this study are publicly available in the Zenodo Repository with accession number 10.5281/zenodo.17366198. The raw counts are publicly available in the Sequence Read Archive database ID SRP635148 under the BioProject ID PRJNA1346442. The accession code for the CD45^+^ dataset repository in GEO is GSE200253. Values for all data points in graphs are reported in the Supporting Data Values file.

## Author contributions

MD, GC, and GT were responsible for patient recruitment and collection of biological specimens and contributed to the study design. DS, VC, EB, RG, SC, JZ, and FS processed samples and conducted experiments. PM, ST, RC, RP, PK, AD, and SNM analyzed the scRNA-Seq data and interpreted the results. CP designed and performed the scRNA-Seq sample preparation. JM, DS, EM, AM, CG, and DM interpreted the results. JM, DS, CG, and DM designed and conducted the study and wrote the manuscript. CG and DM directed and supervised the study. JM and DS share first authorship, having contributed equally in conceiving the overall study. The order of the first authors reflects the leadership exerted in the study.

## Funding support

- Italian Association for Cancer Research (AIRC) (AIRC 5x1000 project 21147 to AM; Fellowships for Italy Post-Doc or PhD to DS, SC, and VC).
- European Union – Next Generation EU (PNRR-MAD-2022-12375947 and PNC-E3-2022-23683266 PNC-HLS-DA to CG).
- Italian Ministry of University and Research (PRIN 20174T7NXL and 2022NKEXAT to CG).
- Competitive fellowships awarded from the PhD program of Experimental Medicine at the University of Milan to PM.
- A competitive fellowship awarded from the Data Science in Medicine and Nutrition (DASMEN) PhD program at Humanitas University to ST.
- Purchase of a FACSymphony A5 was defrayed in part by a grant from the Italian Ministry of Health (Agreement 82/2015).
- The publication fee for this work was covered by the Italian Ministry of Health’s “Ricerca Corrente” funding to the IRCCS Humanitas Research Hospital

## Supplementary Material

Supplemental data

Supporting data values

## Version 1 10/28/2025

In-Press Preview

## Version 2 12/15/2025

Electronic publication

## Version 3 07/15/2026

Author affiliation #1 edited for clarity.
