# Supplementary material for: Tissue-specific antitumor NK cell subsets identified in colorectal cancer liver metastases express candidate therapeutic targets
Source: J Clin Invest. 2025 Oct 28;135(24):e190778. doi: 10.1172/JCI190778 (PMC12704330; doi:10.1172/JCI190778)
Supplement: Supplemental data [file jci-135-190778-s014.pdf]

# Tissue-specific anti-tumor NK cell subsets identified in colorectal cancer liver metastases express candidate therapeutic targets

*Joanna Mikulak[1*], Domenico Supino[2*], Paolo Marzano[1,3], Sara Terzoli[1,4], Roberta Carriero[5], Valentina Cazzetta[1], Rocco Piazza[6], Elena Bruni[1], Paolo Kunderfranco[4], Alessia Donato[2], Sarah Natalia Mapelli[2], Roberto Garuti[4], Silvia Carnevale[7], Francesco Scavello[2], Elena Magrini[2], Jelena Zeleznjak[2,8], Clelia Peano[9,10], Matteo Donadon[11], Guido Costa[12], Guido Torzilli[4,12], Alberto Mantovani[2,4,13], Cecilia Garlanda[2,4†] and Domenico Mavilio[1,3†]*

*[1]Unit of Clinical and Experimental Immunology, IRCCS Humanitas Research Hospital, Rozzano, Milan, Italy.*
*[2]Unit of Experimental Immunopathology, IRCCS Humanitas Research Hospital, Rozzano, Milan, Italy.*
*[3]Department of Medical Biotechnology and Translational Medicine, University of Milan, Milan, Italy.*
*[4]Department of Biomedical Sciences, Humanitas University, Pieve Emanuele, Milan, Italy.*
*[5]Bionformatic Unit, IRCCS Humanitas Research Hospital, Rozzano, Milan, Italy.*
*[6]Department of Medicine and Surgery, University of Milan-Bicocca, Monza, Italy.*
*[7]Unit of Innate immunity in inflammation and cancer, IRCCS Humanitas Research Hospital, Rozzano, Milan, Italy.*
*[8]Center for Proteomics, Faculty of Medicine, University of Rijeka, Rijeka, Croatia.*
*[9]Institute of Genetics and Biomedical Research, UoS of Milan, National Research Council, Rozzano, Milan, Italy.*
*[10]Human Technopole, Milan, Italy.*
*[11]Department of Health Sciences, Università del Piemonte Orientale, Novara, Italy; Department of General Surgery, University Maggiore Hospital Della Carità, Novara, Italy.*
*[12]Unit of Hepatobiliary and General Surgery, IRCCS Humanitas Research Hospital, Rozzano, Milan, Italy.*
*[13]The William Harvey Research Institute, Queen Mary University of London, London, United Kingdom.*

*Supplementary figure 1*

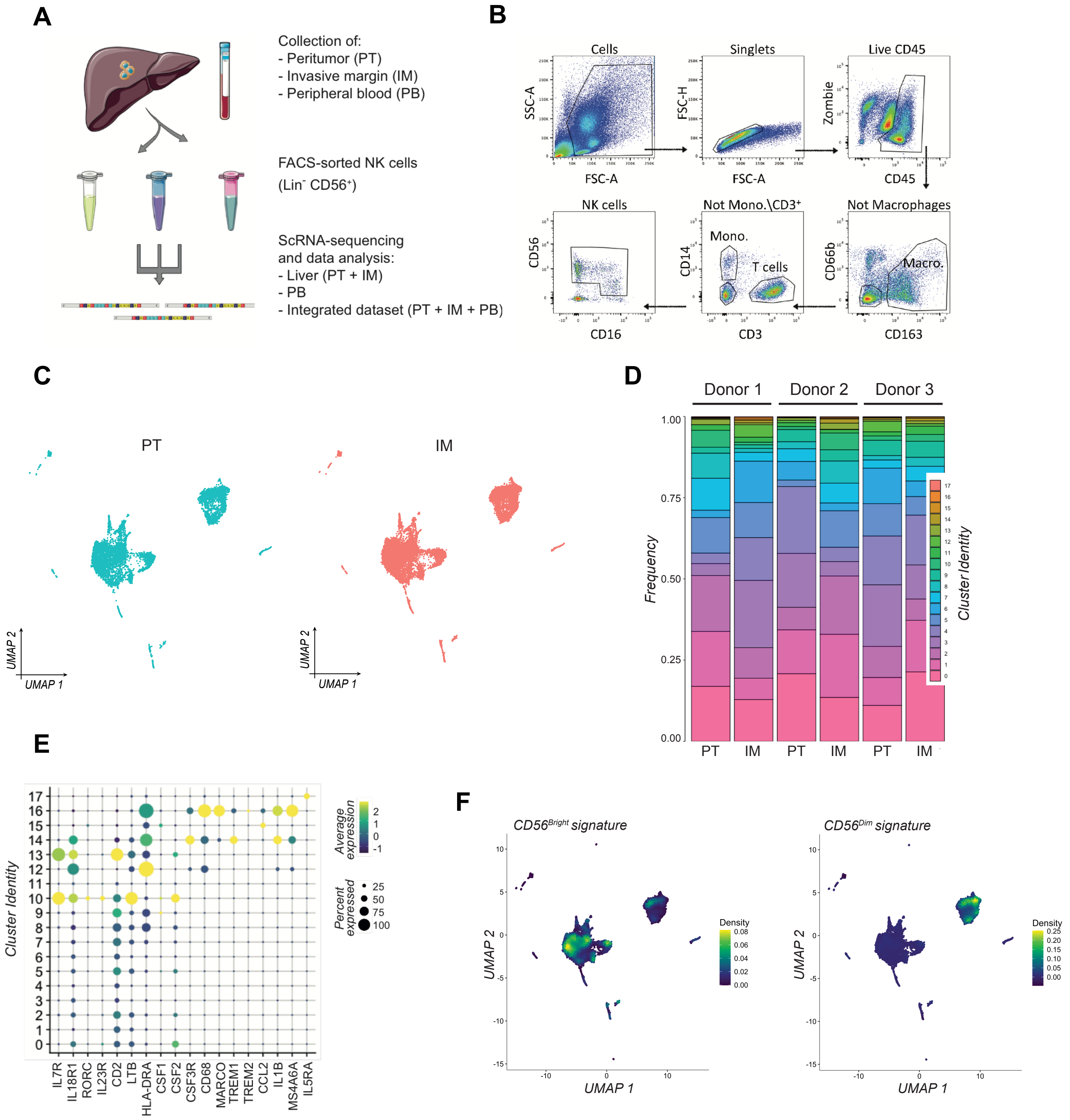

**Supplementary Figure 1. Single cell RNA-seq analysis of NK cells in CRLM samples**

**A.** Schematic overview of the experimental design. **B.** One representative gating strategy (out of 9 samples) of flow cytometry-sorted peripheral blood NK cells to perform scRNA-seq analysis. NK cell purity after FACS-sorting was ≥ 97%. **C.** *UMAP* showing the distribution of $NK_{LR}$ from peritumor (PT) and invasive margin (IM) tissues. **D.** Bar graph showing the frequency (%) of NK cells from the invasive margin (IM) and peritumor (PT) CRLM tissue clusters in each patient (Donor 1-3). Cell numbers were normalized to the total number of cells per tissue and patient. **E.** Dot plot showing the expression of selected myeloid and MAIT cell genes in all $NK_{LR}$ cells. **F.** Kernel density of the $CD56^{Bright}$ and $CD56^{Dim}$ signature scores embedded on UMAP plot.

Supplementary Figure 2

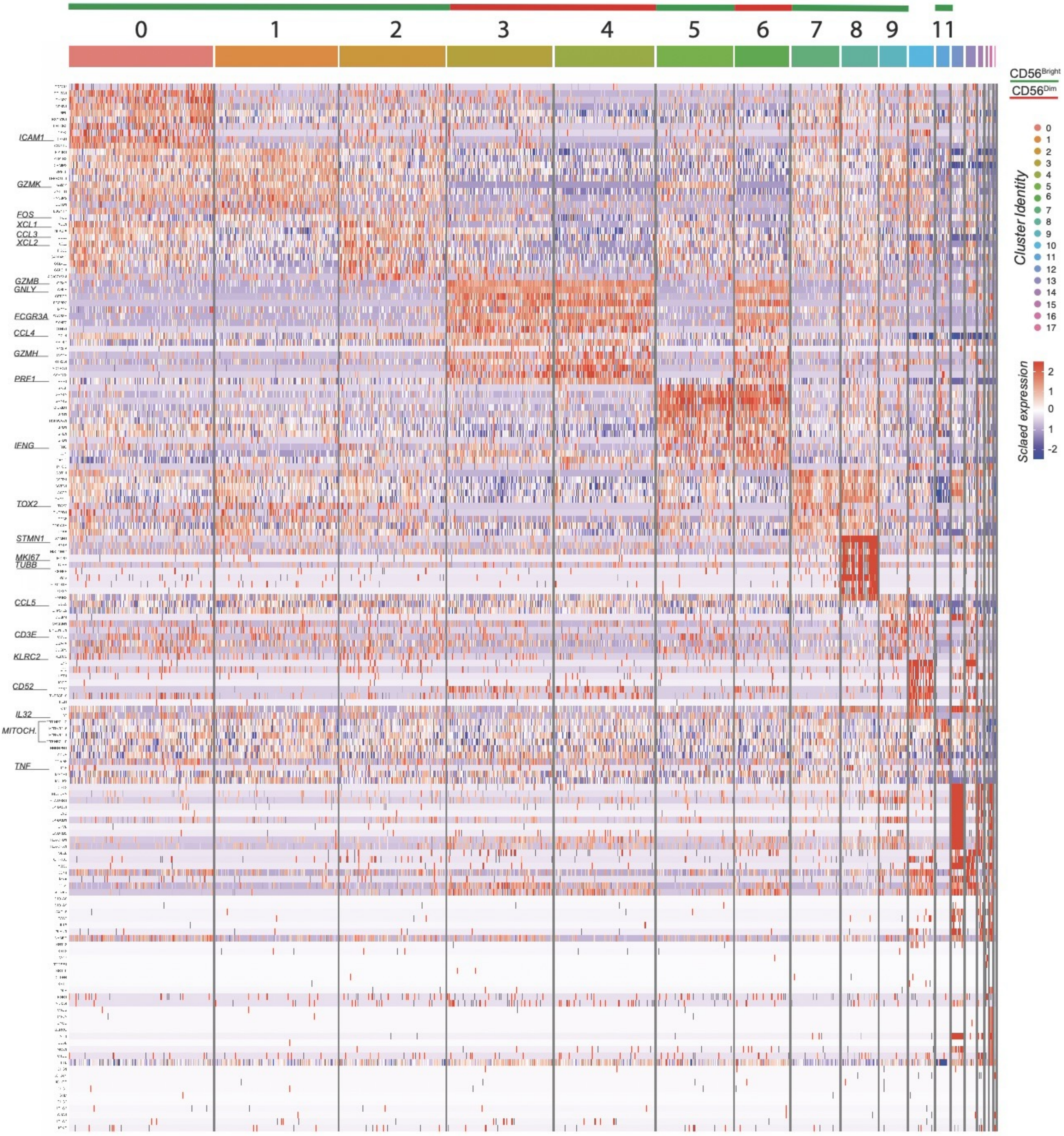

**Supplementary Figure 2. Heatmap of the top 10 DEGs identified in each tissue $NK_{LR}$ cell cluster**

$NK_{LR}$ cell cluster characterization. The heatmap shows the scaled average expression of the top 10 DEGs (rows) for each identified $NK_{LR}$ cell cluster shown in Figure 1A. $CD56^{Bright}$ and $CD56^{Dim}$ $NK_{LR}$ cell subsets are highlighted in green and red, respectively.

*Supplementary Figure 3*

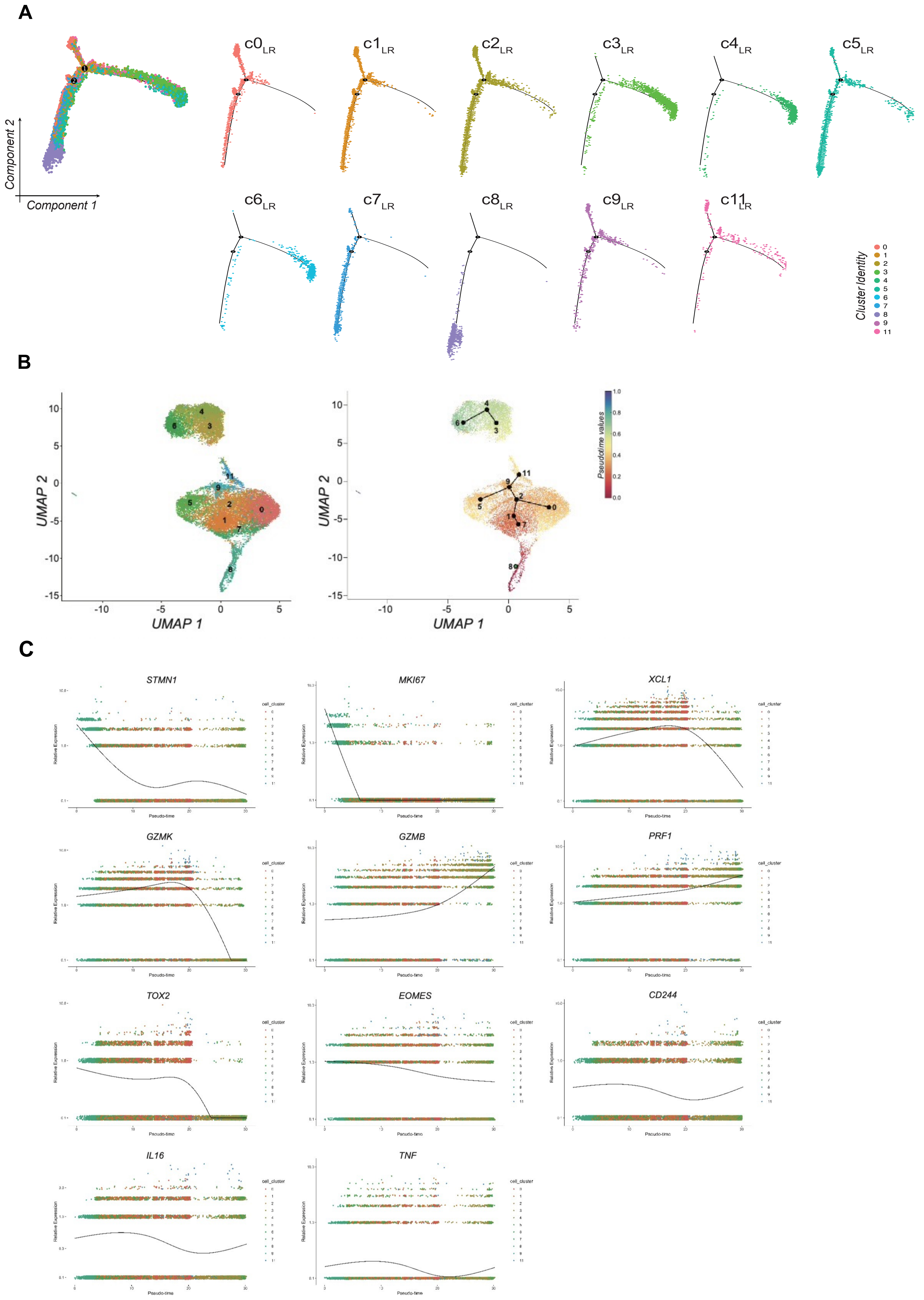

**Supplementary Figure 3. Pseudotime trajectory of $NK_{LR}$**

**A.** Pseudotime trajectory of $NK_{LR}$, where each cell is colored by its cluster identity (left panel). Pseudotime trajectory of each $NK_{LR}$ cell cluster (right panel). **B.** Inferred developmental trajectory with Slingshot of the intrahepatic $NK_{LR}$ cells embedded on the *UMAP.* Left panel, *UMAP* clustering projection; right panel, inferred trajectory displayed on the *UMAP.* **C.** DEGs were selected in cycling (*STMN1*, *MKI67*), $CD56^{Bright}$ (*XCL1*, *GZMK*), $CD56^{Dim}$ (*GZMB*, *PRF1*) $NK_{LR}$ cells or associated with functional features of $NK_{LR}$ from CRLM.

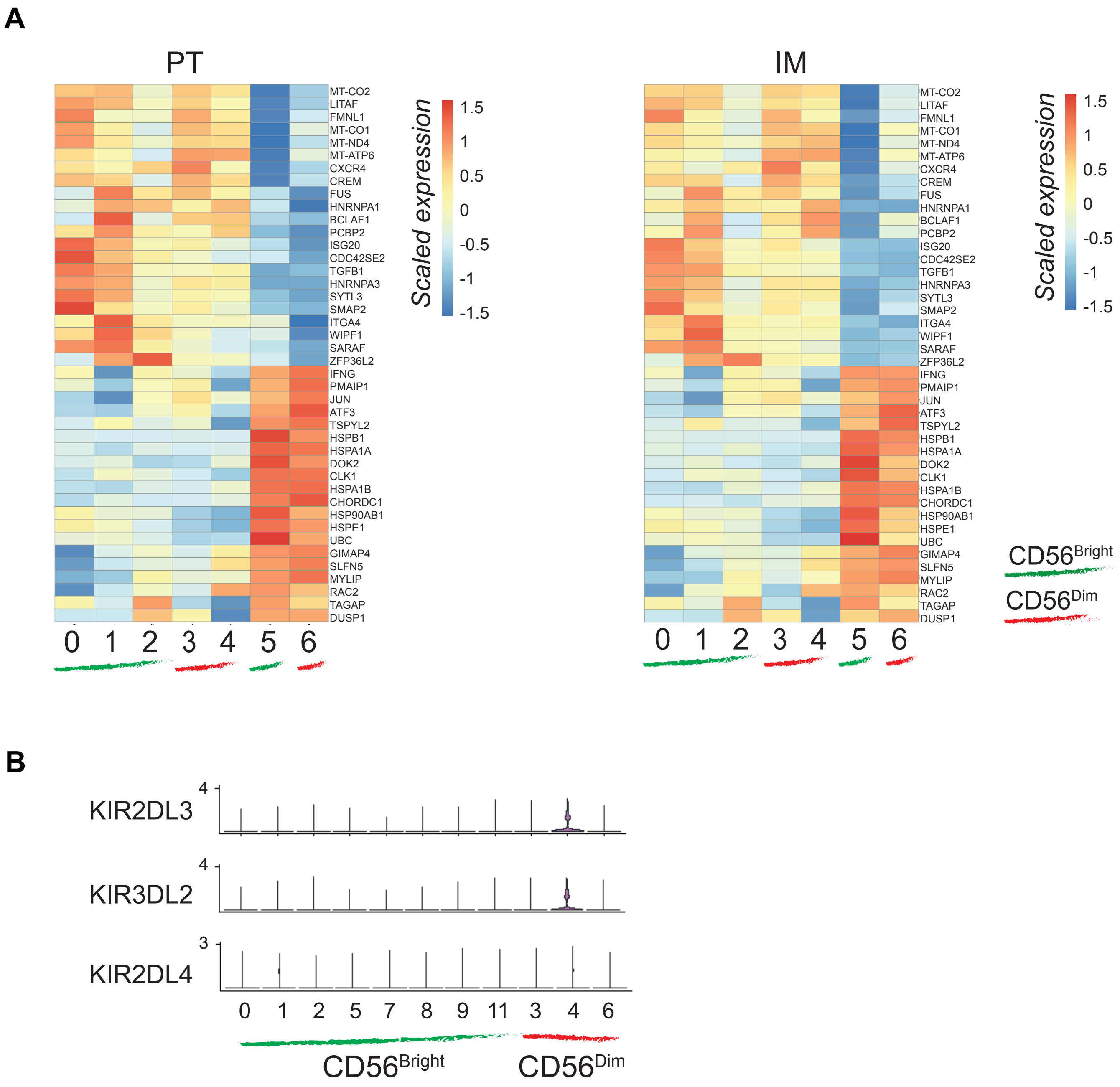
A
PT
IM
MT-CO2
LITAF
FMNL1
MT-CO1
MT-ND4
MT-ATP6
CXCR4
CREM
FUS
HNRNPA1
BCLAF1
PCBP2
ISG20
CDC42SE2
TGFB1
HNRNPA3
SYTL3
SMAP2
ITGA4
WIPF1
SARAF
ZFP36L2
IFNG
PMAIP1
JUN
ATF3
TSPYL2
HSPB1
HSPA1A
DOK2
CLK1
HSPA1B
CHORDC1
HSP90AB1
HSPE1
UBC
GIMAP4
SLFN5
MYLIP
RAC2
TAGAP
DUSP1
0 1 2 3 4 5 6
Scaled expression
1.5
1
0.5
0
-0.5
-1
-1.5
CD56Bright
CD56Dim
B
KIR2DL3
KIR3DL2
KIR2DL4
4
4
3
0 1 2 5 7 8 9 11 3 4 6
CD56Bright
CD56Dim

**Supplementary Figure 4. Shared *IFNG*$^{high}$ signature gene profile among $NK_{LR}$ cell subsets**

**A.** Heatmap showing *IFNG*$^{high}$ signature gene expression in tissue identified $NK_{LR}$ cell clusters for peritumor (PT) (left panel) and invasive margin (IM) (right panel). **B.** Violin plot displaying the expression of selected KIRs. $CD56^{Bright}$ and $CD56^{Dim}$ $NK_{LR}$ cell subsets are highlighted in green and red, respectively.

# Supplementary Figure 5

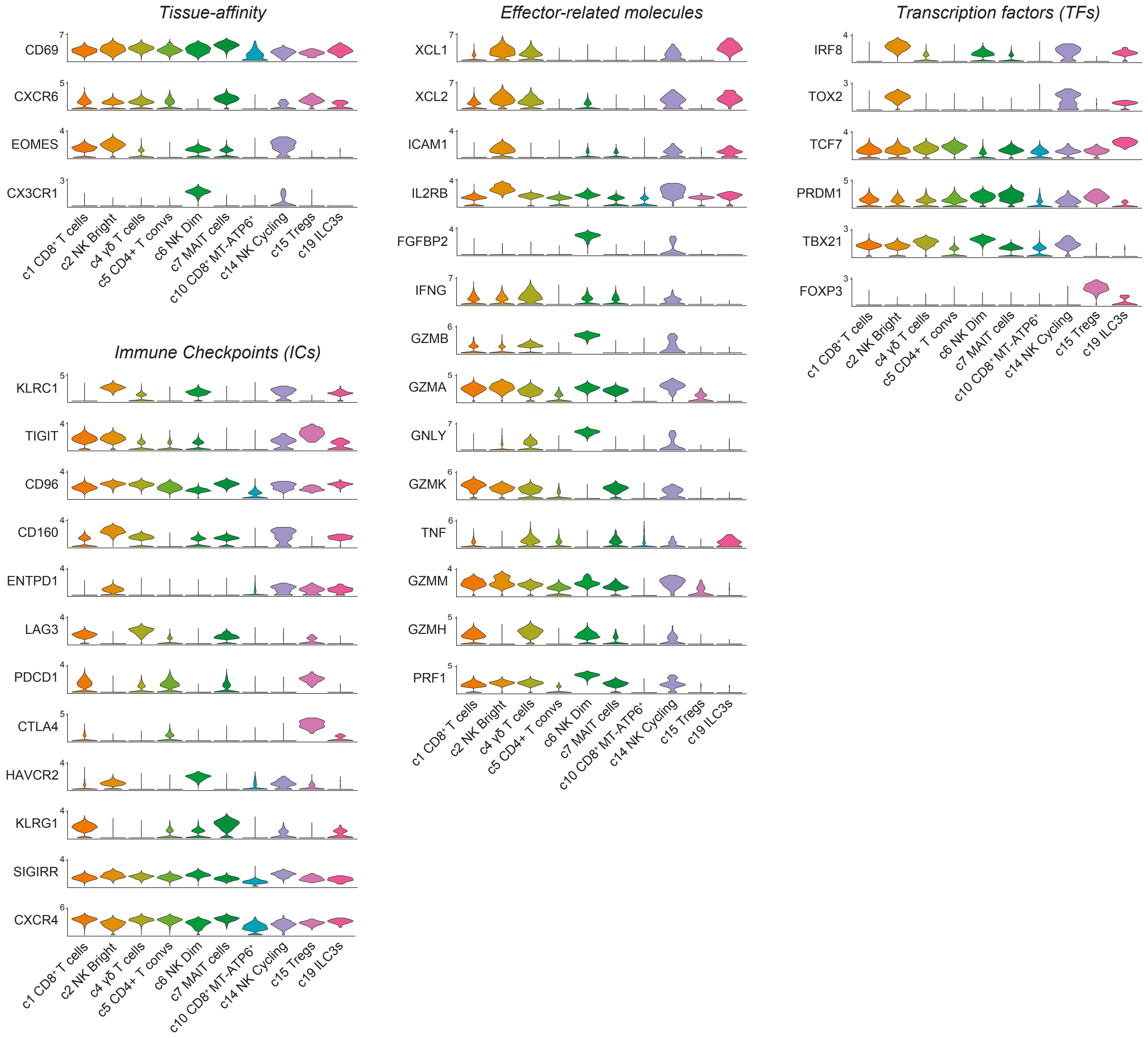

**Supplementary Figure 5. Comparison of hepatic NK cells, T cells and ILC3 in CRLM tissue.**

Violin plots of selected genes across the intrahepatic lymphocyte clusters

*Supplementary Figure 6*

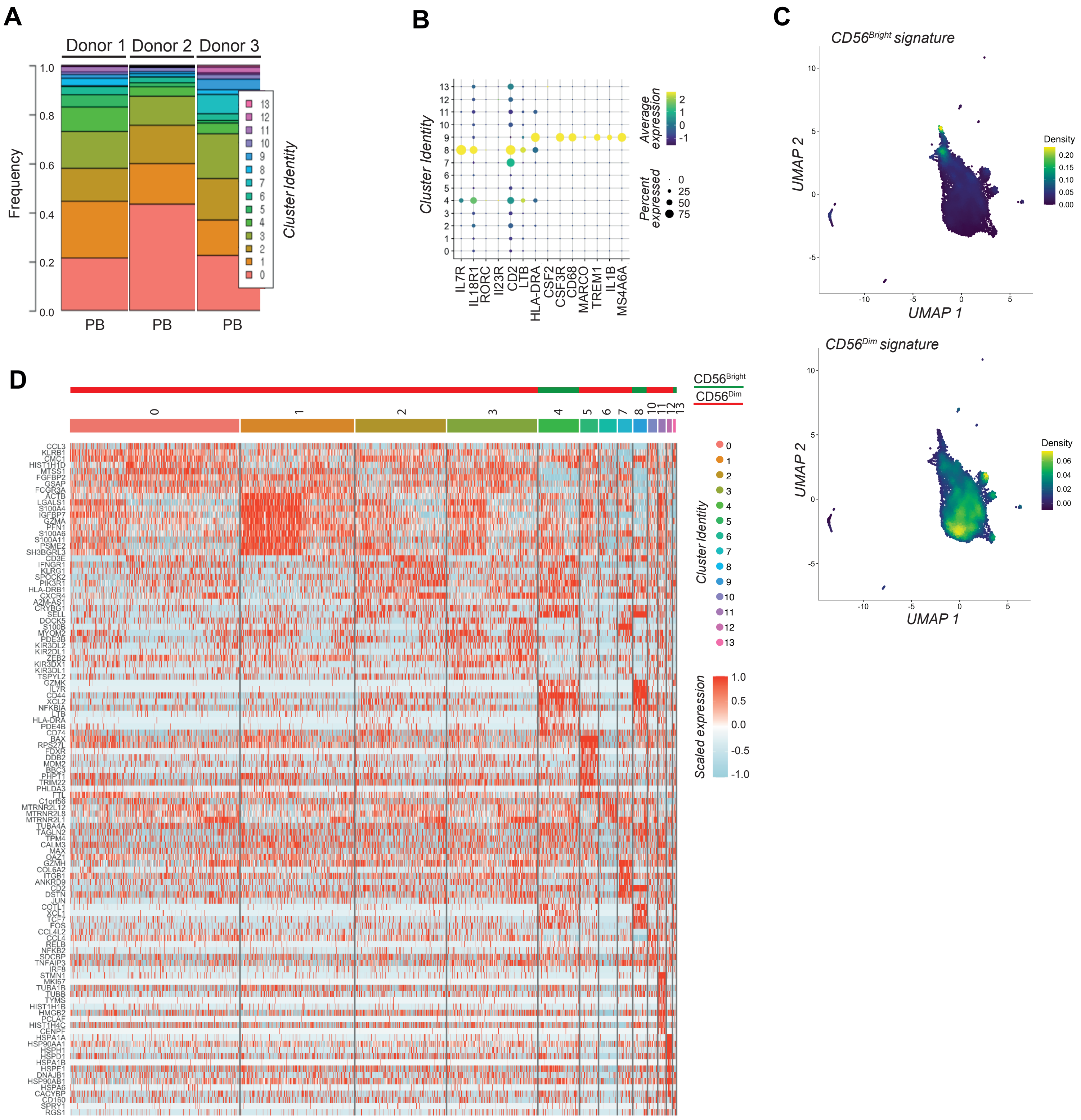

**Supplementary Figure 6. Single cell RNA-seq analysis of blood $NK_{PB}$ cells in CRLM patients**

**A.** Blood $NK_{PB}$ cell cluster characterization. Bar graph representing the frequency (%) of each $NK_{PB}$ cell cluster for each CRLM patient. Cell numbers were normalized to the total number of cells per patient. **B.** Dotplot showing the expression of selected myeloid and MAIT cell genes. **C.** Kernel density of the $CD56^{Bright}$ and $CD56^{Dim}$ signature scores embedded on UMAP plot. **D.** Heatmap showing the scaled average expression of the top 10 DEGs (rows) for each identified $NK_{PB}$ cell cluster shown in Figure 5A. $CD56^{Bright}$ and $CD56^{Dim}$ $NK_{PB}$ cell subsets are highlighted in green and red, respectively.

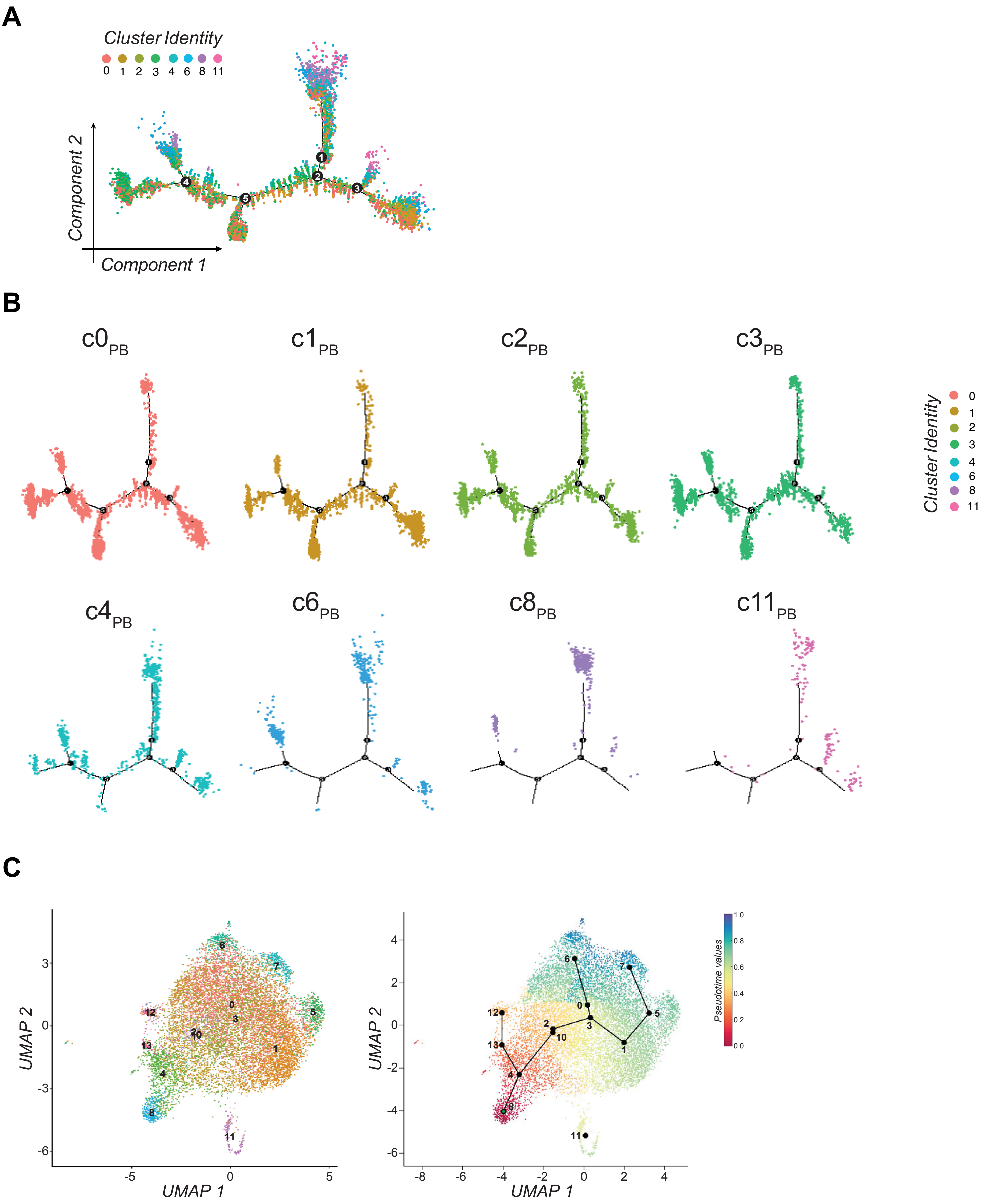
A
Cluster Identity
0 1 2 3 4 6 8 11
Component 2
Component 1
B
c0PB
c1PB
c2PB
c3PB
c4PB
c6PB
c8PB
c11PB
Cluster Identity
0
1
2
3
4
6
8
11
C
UMAP 2
UMAP 1
Pseudotime values
1.0
0.8
0.6
0.4
0.2
0.0

**Supplementary Figure 7. Pseudotime trajectory analysis of blood $NK_{PB}$ cells.**

**A.** Pseudotime trajectory of merged $NK_{PB}$ cell clusters. **B.** Pseudotime trajectory of each $NK_{PB}$ cell cluster. Each cell is colored by its cluster identity. **C.** Inferred developmental trajectory of the circulating NK cells computed with Slingshot algorithm. Left panel, *UMAP* clustering projection; right panel, inferred trajectory displayed on the *UMAP*.

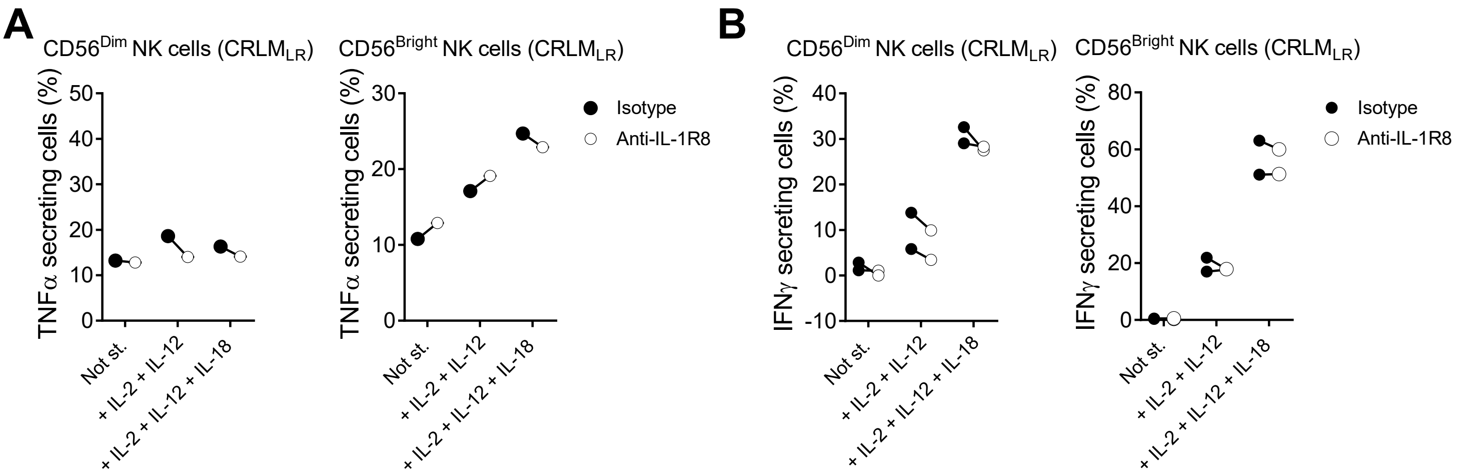
A
CD56Dim NK cells (CRLMLR)
TNFα secreting cells (%)
0
10
20
30
40
50
Not st.
+ IL-2 + IL-12
+ IL-2 + IL-12 + IL-18
CD56Bright NK cells (CRLMLR)
TNFα secreting cells (%)
0
10
20
30
Isotype
Anti-IL-1R8
B
CD56Dim NK cells (CRLMLR)
IFNγ secreting cells (%)
-10
0
10
20
30
40
CD56Bright NK cells (CRLMLR)
IFNγ secreting cells (%)
0
20
40
60
80
Isotype
Anti-IL-1R8

**Supplementary Figure 8. Not-responder patients stimulated with anti-hIL-1R8 mAb**

**A-B.** Evaluation of TNFα (A) and IFNγ (B) in $CD56^{Dim}$ and $CD56^{Bright}$ NK cells from CRLM patients stimulated with an anti-hIL-1R8 mAb or its isotype control.

**Supplementary Table 1**
**Monoclonal antibodies (mAbs) used for flow cytometry analysis.**

| Marker | Fluorochrome | Clone | Company | Catalogue number |
|---|---|---|---|---|
| CD3 | BUV661 | UCHT1 | BD | 565065 |
| CD16 | BUV737 | 3G8 | BD | 564434 |
| CD56 | BUV563 | NCAM16.2 | BD | 565704 |
| CXCR4 | BUV395 | 12G5 | BD | 563924 |
| CXCR6 | BV650 | 13B | BD | 743600 |
| CTLA-4 | APC | BNI3 | BD | 560938 |
| ILT-2 | PE-Cy5 | GHI/75 | BD | 551054 |
| Ki67 | PE | B56 | BD | 51-36525X |
| LAG-3 | APC-R700 | T47-530 | BD | 565774 |
| LAIR-1 | PE | DX26 | BD | 550811 |
| TIGIT | BV421 | 741182 | BD | 747844 |
| TIM-3 | BV711 | 7D3 | BD | 565566 |
| CD14 | BV570 | M5E2 | BioLegend | 301832 |
| CD19 | BV570 | HIB19 | BioLegend | 302236 |
| CD39 | PE/Dazzle594 | A1 | BioLegend | 328224 |
| CD96 | PE-Cy7 | NK92.39 | BioLegend | 338415 |
| KLRG1 | BV785 | 2F1/KLRG1 | BioLegend | 138429 |
| IFNγ | PE-Cy7 | B27 | BioLegend | 506518 |
| TNFα | BV785 | Mab11 | BioLegend | 502948 |
| NKG2A | VioBright-FITC | REA110 | Miltenyi | 130105646 |
| IL-1R8 | Biotin | | R&D | BAF990 |

Supplementary Table 2

| NK Dim score | NK Bright score | Cell cycle score | |
|---|---|---|---|
| *CD160* | *LTB* | *MCM5* | *NUSAP1* |
| *CTSD* | *FOS* | *PCNA* | *UBE2C* |
| *CCL4* | *IL2RB* | *TYMS* | *BIRC5* |
| *ADGRG1* | *IFITM3* | *FEN1* | *TPX2* |
| *CD38* | *COTL1* | *MCM2* | *TOP2A* |
| *CD247* | *IL7R* | *MCM4* | *NDC80* |
| *CHST2* | *PIK3R1* | *RRM1* | *CKS2* |
| *CX3CR1* | *AREG* | *UNG* | *NUF2* |
| *KLRB1* | *ZFP36L2* | *GINS2* | *CKS1B* |
| *LAIR2* | *DUSP2* | *MCM6* | *MKI67* |
| *IGFBP7* | *CD44* | *CDCA7* | *TMPO* |
| *AKR1C3* | *SELL* | *DTL* | *CENPF* |
| *FGFBP2* | *GPR183* | *PRIM1* | *TACC3* |
| *MYOM2* | *CMC1* | *UHRF1* | *FAM64A* |
| *CLIC3* | *KLRC1* | *HELLS* | *SMC4* |
| *GZMB* | *TCF7* | *RFC2* | *CCNB2* |
| *PRF1* | *TPT1* | *RPA2* | *CKAP2L* |
| *FCER1G* | *XCL2* | *NASP* | *CKAP2* |
| *NKG7* | *XCL1* | *RAD51AP1* | *AURKB* |
| *SPON2* | *GZMK* | *GMNN* | *BUB1* |
| *LGALS1* | *CCL3* | *WDR76* | *KIF11* |
| *GZMH* | *ZNF331* | *SLBP* | *ANP32E* |
| *S100A4* | *TOX2* | *CCNE2* | *TUBB4B* |
| *PRSS23* | *NR4A2* | *UBR7* | *GTSE1* |
| *GNLY* | *CD160* | *POLD3* | *KIF20B* |
| *S100A6* | *SERPINE2* | *MSH2* | *HJURP* |
| *SYNE2* | *FAM177A1* | *ATAD2* | *CDCA3* |
| *EMP3* | *ABHD15* | *RAD51* | *HN1* |
| *CD99* | *LEPROTL1* | *RRM2* | *CDC20* |
| *KIR2DL3* | *CD27* | *CDC45* | *TTK* |
| *S1PR5* | *CXCR6* | *CDC6* | *CDC25C* |
| *KIR2DL1* | *CD69* | *EXO1* | *KIF2C* |
| *FCRL6* | *ADGRG3* | *TIPIN* | *RANGAP1* |
| *HOPX* | *SPRY1* | *DSCC1* | *NCAPD2* |
| *GTF3C1* | *ALOX5AP* | *BLM* | *DLGAP5* |
| | *TIGIT* | *CASP8AP2* | *CDCA2* |
| | *IFRD1* | *USP1* | *CDCA8* |
| | *IRF8* | *CLSPN* | *ECT2* |
| | *TSC22D3* | *POLA1* | *KIF23* |
| | *KLF6* | *CHAF1B* | *HMMR* |
| | *ELF1* | *BRIP1* | *AURKA* |
| | *JUND* | *E2F8* | *PSRC1* |
| | *STAT4* | *HMGB2* | *ANLN* |
| | | *CDK1* | *LBR* |
| | | | *CKAP5* |
| | | | *CENPE* |
| | | | *CTCF* |
| | | | *NEK2* |
| | | | *G2E3* |
| | | | *GAS2L3* |
| | | | *CBX5* |
| | | | *CENPA* |
